# Supplementary material for: Gut microbiota-derived extracellular vesicles form a distinct entity from gut microbiota
Source: mSystems. 2025 Apr 29;10(5):e00311-25. doi: 10.1128/msystems.00311-25 (PMC12090791; doi:10.1128/msystems.00311-25)
Supplement: Supplemental figures, part 1 — Figures S1 to S11. [file msystems.00311-25-s0001.pdf]

Supplemental figures (1-11)

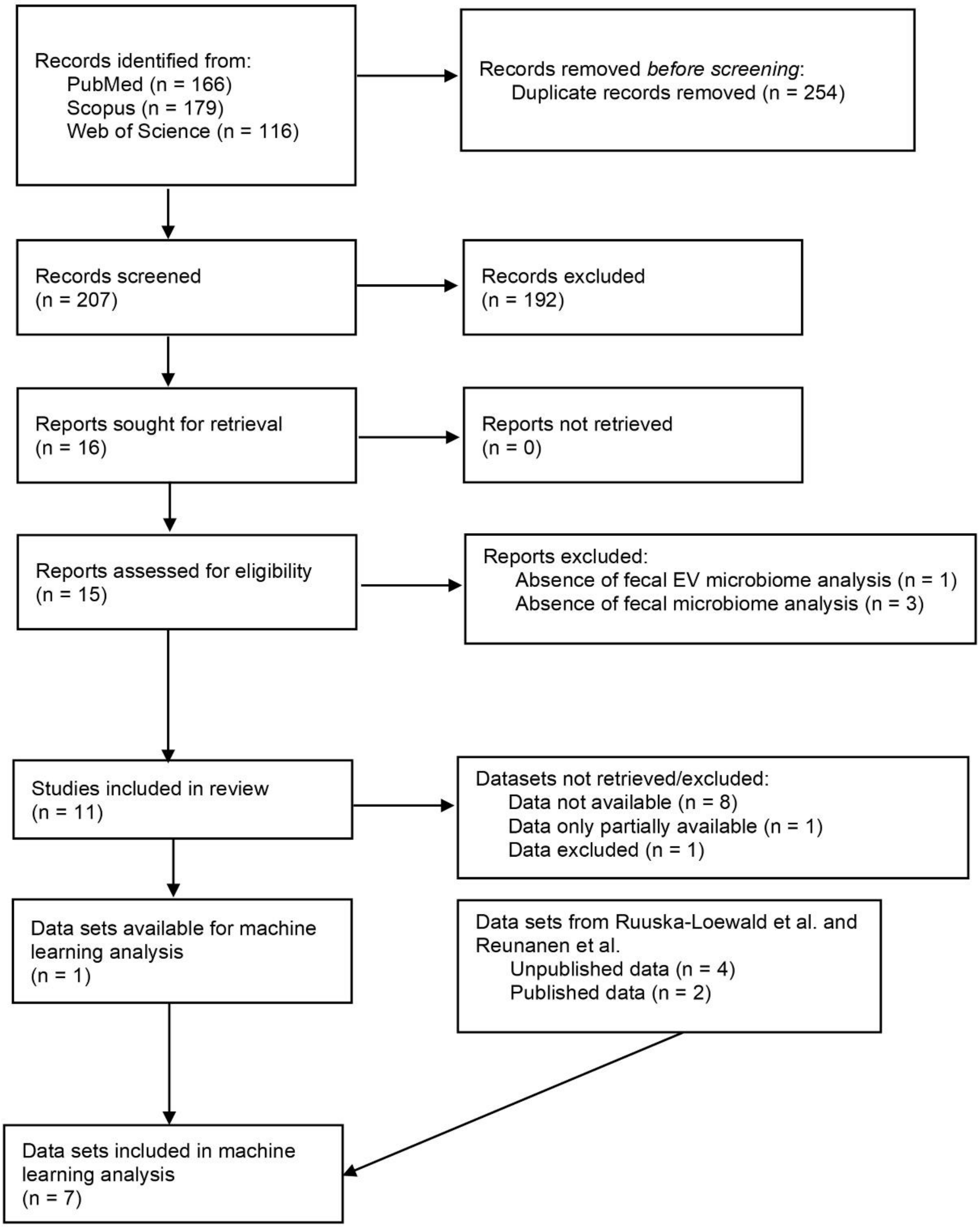

Supplemental figure 1. Preferred Reporting Items for Systematic Reviews and Meta-Analyses (PRISMA) flowchart of the systematic review process used to identify published 16S rRNA gene sequencing datasets.

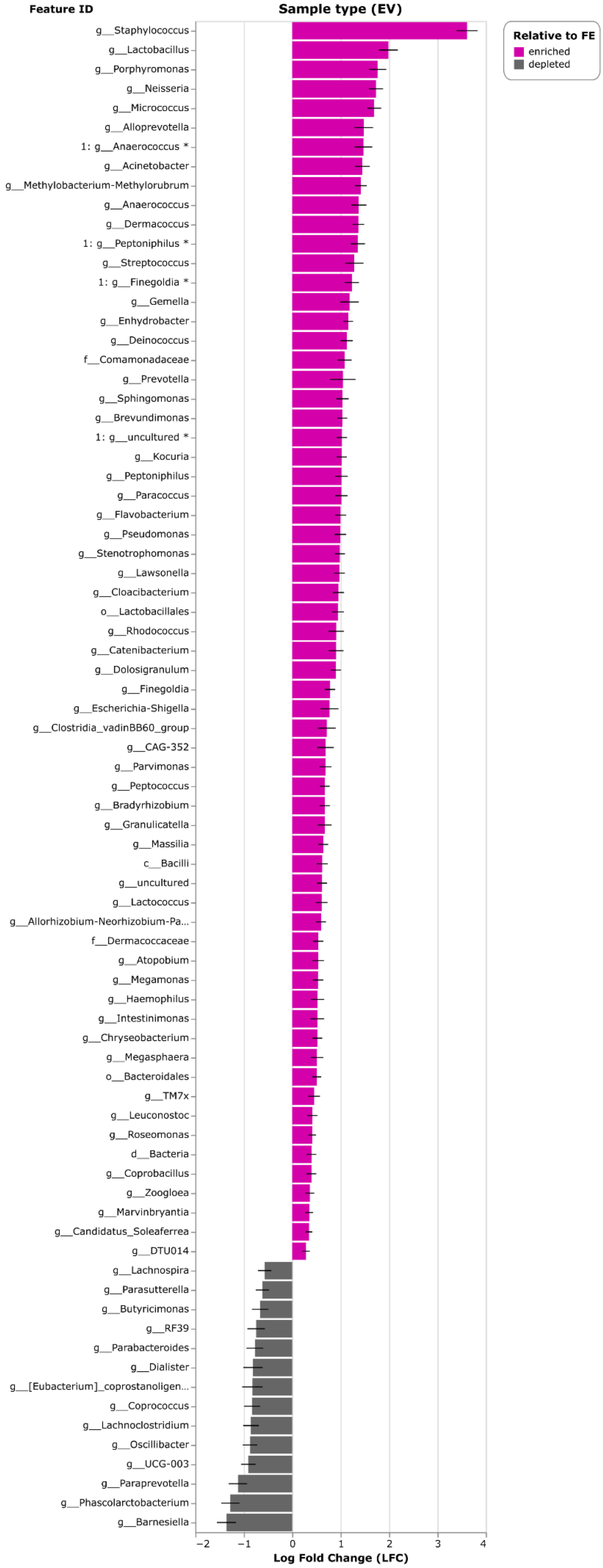
Feature ID
Sample type (EV)
Relative to FE
enriched
depleted
g__Staphylococcus
g__Lactobacillus
g__Porphyromonas
g__Neisseria
g__Micrococcus
g__Alloprevotella
1: g__Anaerococcus *
g__Acinetobacter
g__Methylobacterium-Methylorubrum
g__Anaerococcus
g__Dermacoccus
1: g__Peptoniphilus *
g__Streptococcus
1: g__Finegoldia *
g__Gemella
g__Enhydrobacter
g__Deinococcus
f__Comamonadaceae
g__Prevotella
g__Sphingomonas
g__Brevundimonas
1: g__uncultured *
g__Kocuria
g__Peptoniphilus
g__Paracoccus
g__Flavobacterium
g__Pseudomonas
g__Stenotrophomonas
g__Lawsonella
g__Cloacibacterium
o__Lactobacillales
g__Rhodococcus
g__Catenibacterium
g__Dolosigranulum
g__Finegoldia
g__Escherichia-Shigella
g__Clostridia_vadinBB60_group
g__CAG-352
g__Parvimonas
g__Peptococcus
g__Bradyrhizobium
g__Granulicatella
g__Massilia
c__Bacilli
g__uncultured
g__Lactococcus
g__Allorhizobium-Neorhizobium-Pa...
f__Dermacoccaceae
g__Atopobium
g__Megamonas
g__Haemophilus
g__Intestinimonas
g__Chryseobacterium
g__Megasphaera
o__Bacteroidales
g__TM7x
g__Leuconostoc
g__Roseomonas
d__Bacteria
g__Coprobacillus
g__Zoogloea
g__Marvinbryantia
g__Candidatus_Soleaferrea
g__DTU014
g__Lachnospira
g__Parasutterella
g__Butyricimonas
g__RF39
g__Parabacteroides
g__Dialister
g__[Eubacterium]_coprostanoligen...
g__Coprococcus
g__Lachnoclostridium
g__Oscillibacter
g__UCG-003
g__Paraprevotella
g__Phascolarctobacterium
g__Barnesiella
−2
−1
0
1
2
3
4
Log Fold Change (LFC)

Supplemental figure 2. Differentially abundant genera between gut microbiota-derived EV and fecal samples pooled from all the datasets with 95% confidence intervals for the mean differential abundance for all genera. Genera with positive LFC values (pink) are enriched, and genera with negative LFC values (grey) are depleted in the gut microbiota-derived EVs compared to the fecal samples.

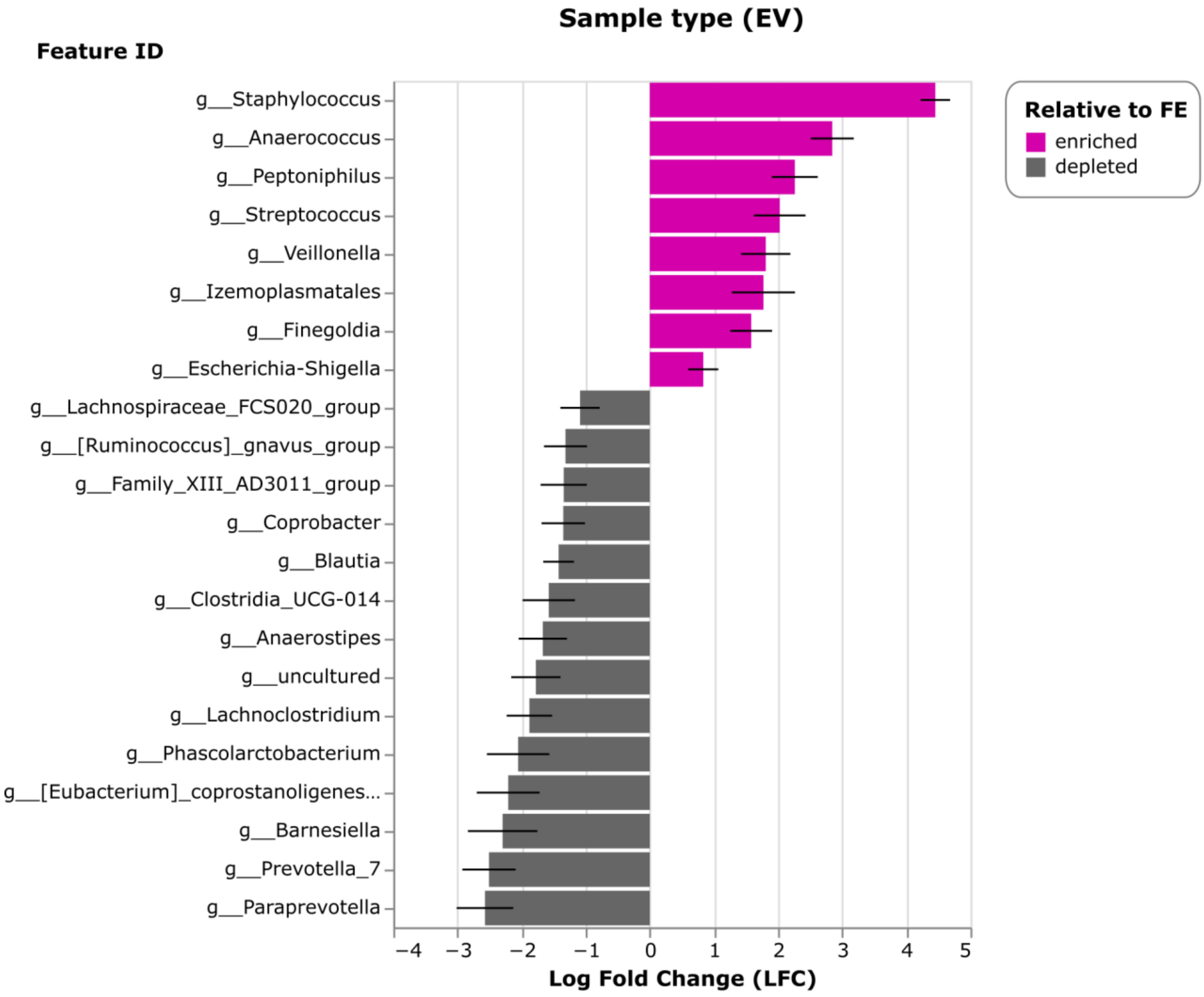

Supplemental figure 3. Differentially abundant genera between gut microbiota-derived EV and fecal samples in the lymphoma (all) dataset, with 95% confidence intervals for the mean differential abundance for all genera. Genera with positive LFC values (pink) are enriched, and genera with negative LFC values (grey) are depleted in the gut microbiota-derived EVs compared to the fecal samples.

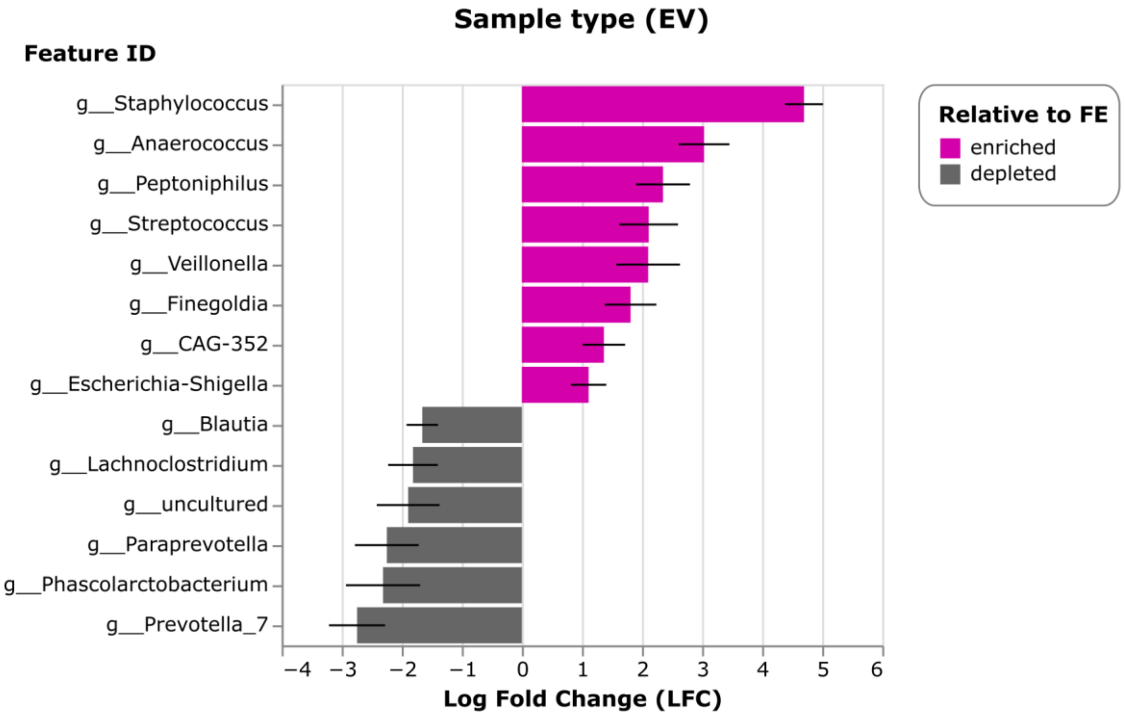

Supplemental figure 4. Differentially abundant genera between gut microbiota-derived EV and fecal samples in the lymphoma (DLBL) dataset, with 95% confidence intervals for the mean differential abundance for all genera. Genera with positive LFC values (pink) are enriched, and genera with negative LFC values (grey) are depleted in the gut microbiota-derived EVs compared to the fecal samples.

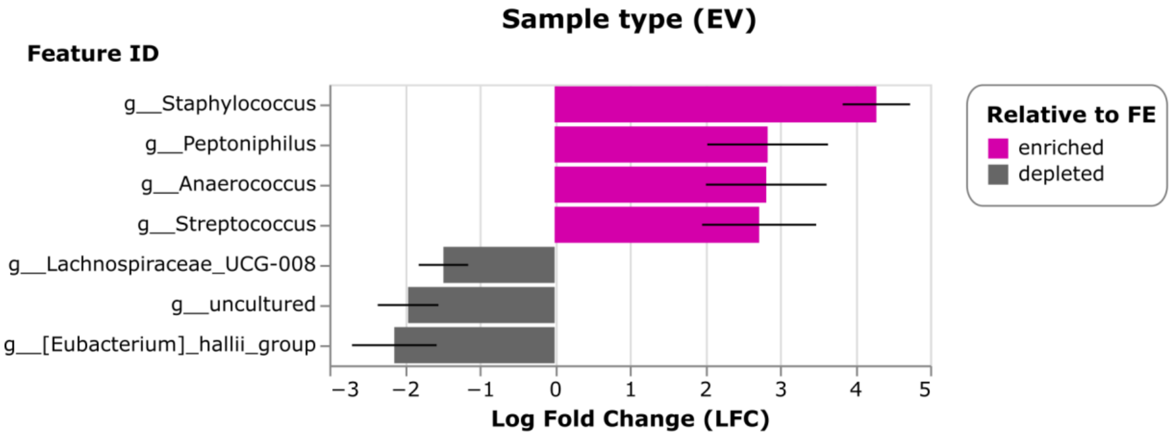

Supplemental figure 5. Differentially abundant genera between gut microbiota-derived EV and fecal samples in the lymphoma (Hodgkin) dataset, with 95% confidence intervals for the mean differential abundance for all genera. Genera with positive LFC values (pink) are enriched, and genera with negative LFC values (grey) are depleted in the gut microbiota-derived EVs compared to the fecal samples.

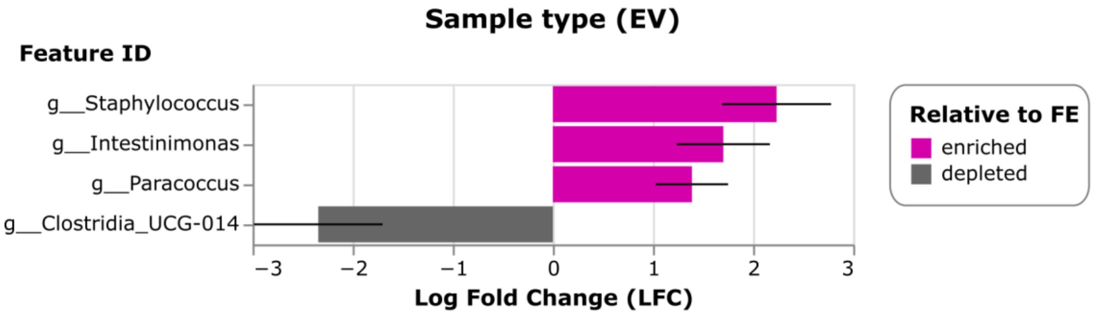

Supplemental figure 6. Differentially abundant genera between gut microbiota-derived EV and fecal samples in the Parkinson's disease dataset, with 95% confidence intervals for the mean differential abundance for all genera. Genera with positive LFC values (pink) are enriched, and genera with negative LFC values (grey) are depleted in the gut microbiota-derived EVs compared to the fecal samples.

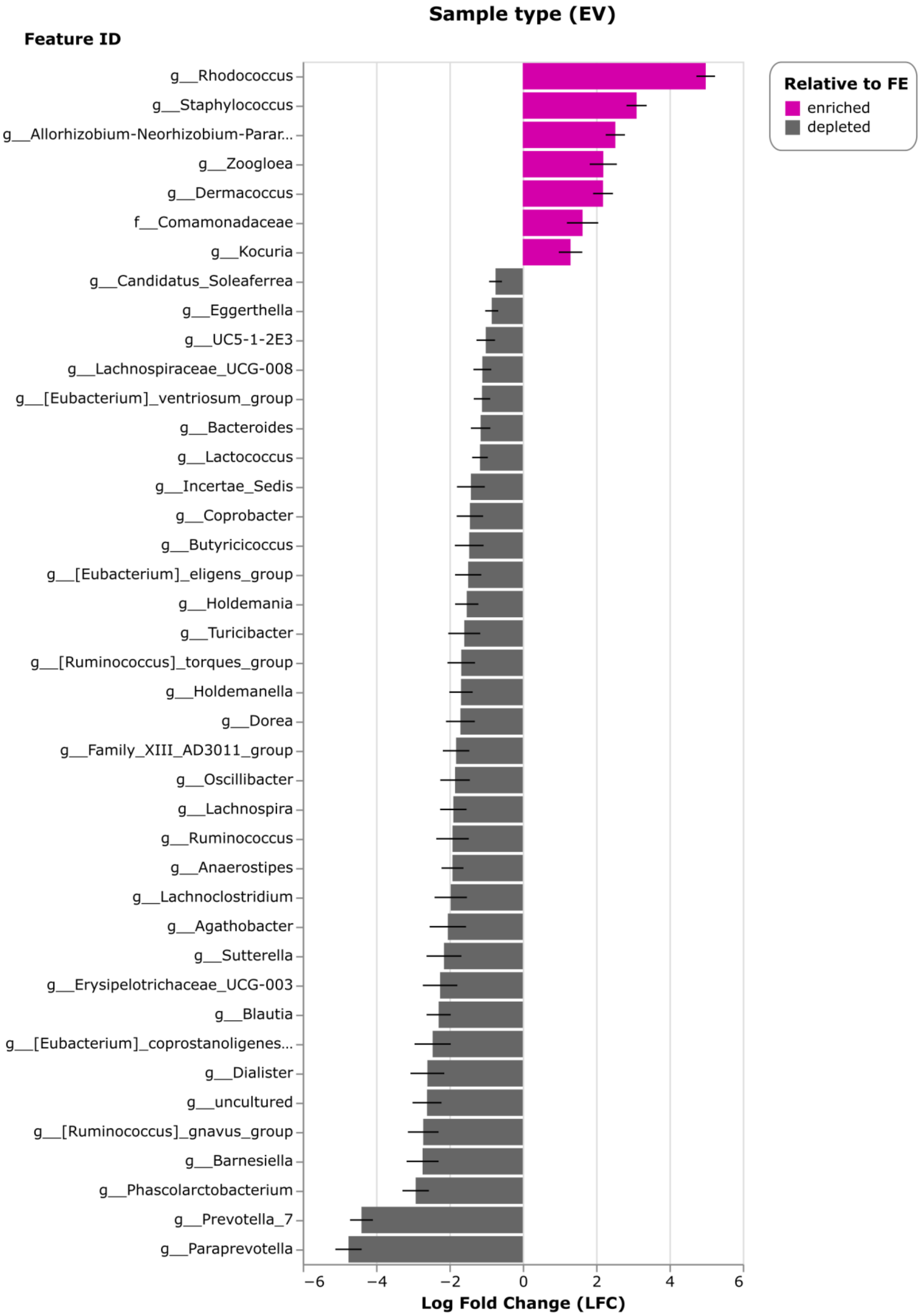
Sample type (EV)
Feature ID
g__Rhodococcus
g__Staphylococcus
g__Allorhizobium-Neorhizobium-Parar...
g__Zoogloea
g__Dermacoccus
f__Comamonadaceae
g__Kocuria
g__Candidatus_Soleaferrea
g__Eggerthella
g__UC5-1-2E3
g__Lachnospiraceae_UCG-008
g__[Eubacterium]_ventriosum_group
g__Bacteroides
g__Lactococcus
g__Incertae_Sedis
g__Coprobacter
g__Butyricicoccus
g__[Eubacterium]_eligens_group
g__Holdemania
g__Turicibacter
g__[Ruminococcus]_torques_group
g__Holdemanella
g__Dorea
g__Family_XIII_AD3011_group
g__Oscillibacter
g__Lachnospira
g__Ruminococcus
g__Anaerostipes
g__Lachnoclostridium
g__Agathobacter
g__Sutterella
g__Erysipelotrichaceae_UCG-003
g__Blautia
g__[Eubacterium]_coprostanoligenes...
g__Dialister
g__uncultured
g__[Ruminococcus]_gnavus_group
g__Barnesiella
g__Phascolarctobacterium
g__Prevotella_7
g__Paraprevotella
−6
−4
−2
0
2
4
6
Log Fold Change (LFC)
Relative to FE
enriched
depleted

Supplemental figure 7. Differentially abundant genera between gut microbiota-derived EV and fecal samples in the solid tumor dataset with 95% confidence intervals for the mean differential abundance for all genera. Genera with positive LFC values (pink) are enriched, and genera with negative LFC values (grey) are depleted in the gut microbiota-derived EVs compared to the fecal samples.

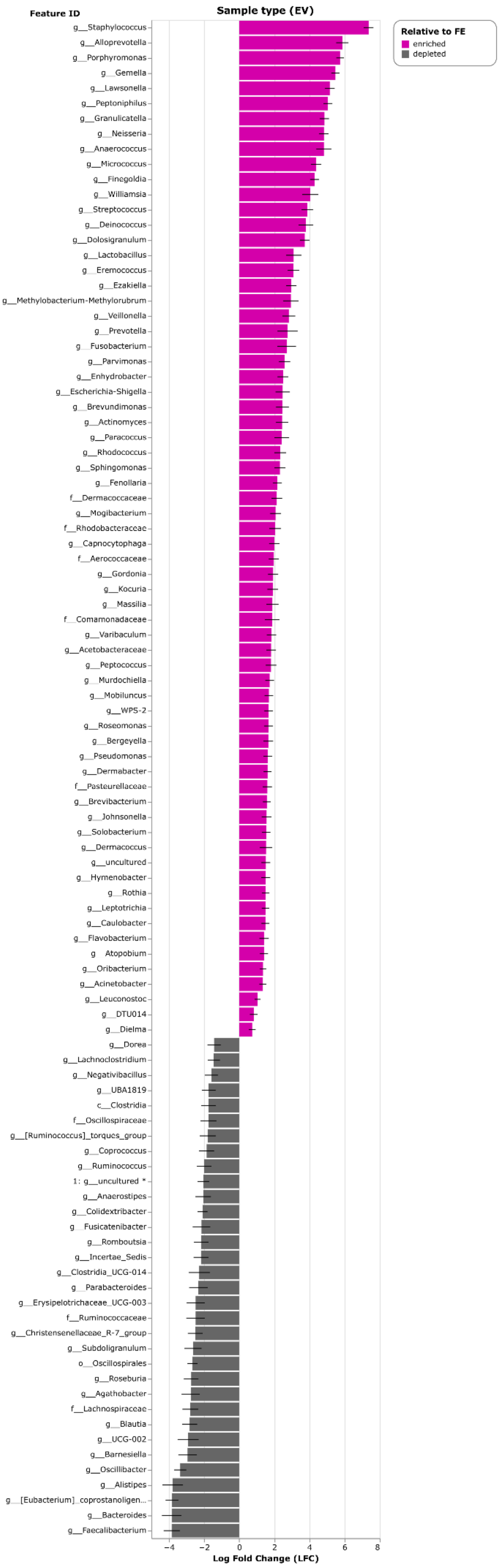

Feature ID
Sample type (EV)
Relative to FE
enriched
depleted
g__Staphylococcus
g__Alloprevotella
g__Porphyromonas
g__Gemella
g__Lawsonella
g__Peptoniphilus
g__Granulicatella
g__Neisseria
g__Anaerococcus
g__Micrococcus
g__Finegoldia
g__Williamsia
g__Streptococcus
g__Deinococcus
g__Dolosigranulum
g__Lactobacillus
g__Eremococcus
g__Ezakiella
g__Methylobacterium-Methylorubrum
g__Veillonella
g__Prevotella
g__Fusobacterium
g__Parvimonas
g__Enhydrobacter
g__Escherichia-Shigella
g__Brevundimonas
g__Actinomyces
g__Paracoccus
g__Rhodococcus
g__Sphingomonas
g__Fenollaria
f__Dermacoccaceae
g__Mogibacterium
f__Rhodobacteraceae
g__Capnocytophaga
f__Aerococcaceae
g__Gordonia
g__Kocuria
g__Massilia
f__Comamonadaceae
g__Varibaculum
g__Acetobacteraceae
g__Peptococcus
g__Murdochiella
g__Mobiluncus
g__WPS-2
g__Roseomonas
g__Bergeyella
g__Pseudomonas
g__Dermabacter
f__Pasteurellaceae
g__Brevibacterium
g__Johnsonella
g__Solobacterium
g__Dermacoccus
g__uncultured
g__Hymenobacter
g__Rothia
g__Leptotrichia
g__Caulobacter
g__Flavobacterium
g__Atopobium
g__Oribacterium
g__Acinetobacter
g__Leuconostoc
g__DTU014
g__Dielma
g__Dorea
g__Lachnoclostridium
g__Negativibacillus
g__UBA1819
c__Clostridia
f__Oscillospiraceae
g__[Ruminococcus]_torques_group
g__Coprococcus
g__Ruminococcus
1: g__uncultured *
g__Anaerostipes
g__Colidextribacter
g__Fusicatenibacter
g__Romboutsia
g__Incertae_Sedis
g__Clostridia_UCG-014
g__Parabacteroides
g__Erysipelotrichaceae_UCG-003
f__Ruminococcaceae
g__Christensenellaceae_R-7_group
g__Subdoligranulum
o__Oscillospirales
g__Roseburia
g__Agathobacter
f__Lachnospiraceae
g__Blautia
g__UCG-002
g__Barnesiella
g__Oscillibacter
g__Alistipes
g__[Eubacterium]_coprostanoligen...
g__Bacteroides
g__Faecalibacterium
−4
−2
0
2
4
6
8
Log Fold Change (LFC)

Supplemental figure 8. Differentially abundant genera between gut microbiota-derived EV and fecal samples in the pregnancy dataset, with 95% confidence intervals for the mean differential abundance for all genera. Genera with positive LFC values (pink) are enriched, and genera with negative LFC values (grey) are depleted in the gut microbiota-derived EVs compared to the fecal samples.

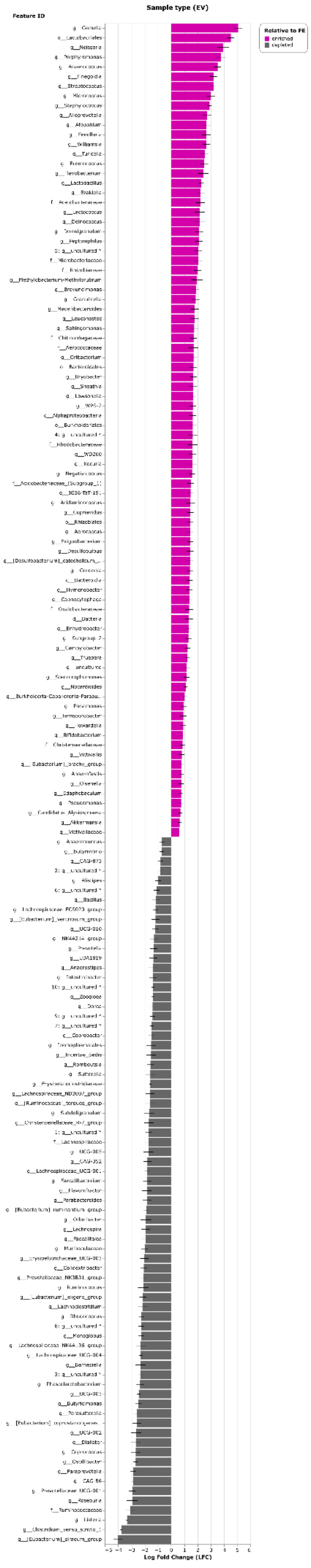

Sample type (EV)
Feature ID
Relative to FE
enriched
depleted
Log Fold Change (LFC)

Supplemental figure 9. Differentially abundant genera between gut microbiota-derived EV and fecal samples in the obesity dataset, with 95% confidence intervals for the mean differential abundance for all genera. Genera with positive LFC values (pink) are enriched, and genera with negative LFC values (grey) are depleted in the gut microbiota-derived EVs compared to the fecal samples.

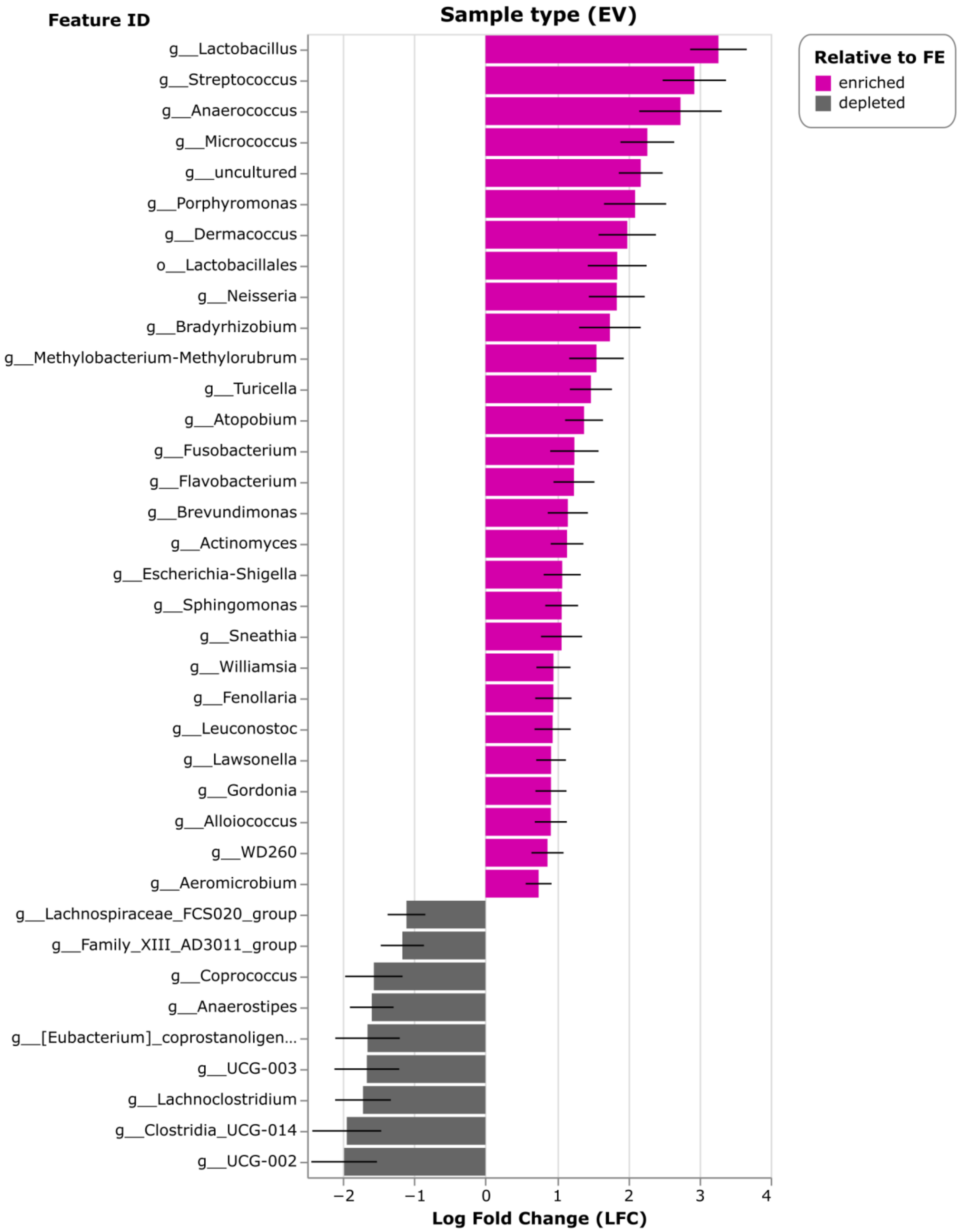

Supplemental figure 10. Differentially abundant genera between gut microbiota-derived EV and fecal samples in the non-disease dataset, with 95% confidence intervals for the mean differential abundance for all genera. Genera with positive LFC values (pink) are enriched, and genera with negative LFC values (grey) are depleted in the gut microbiota-derived EVs compared to the fecal samples.

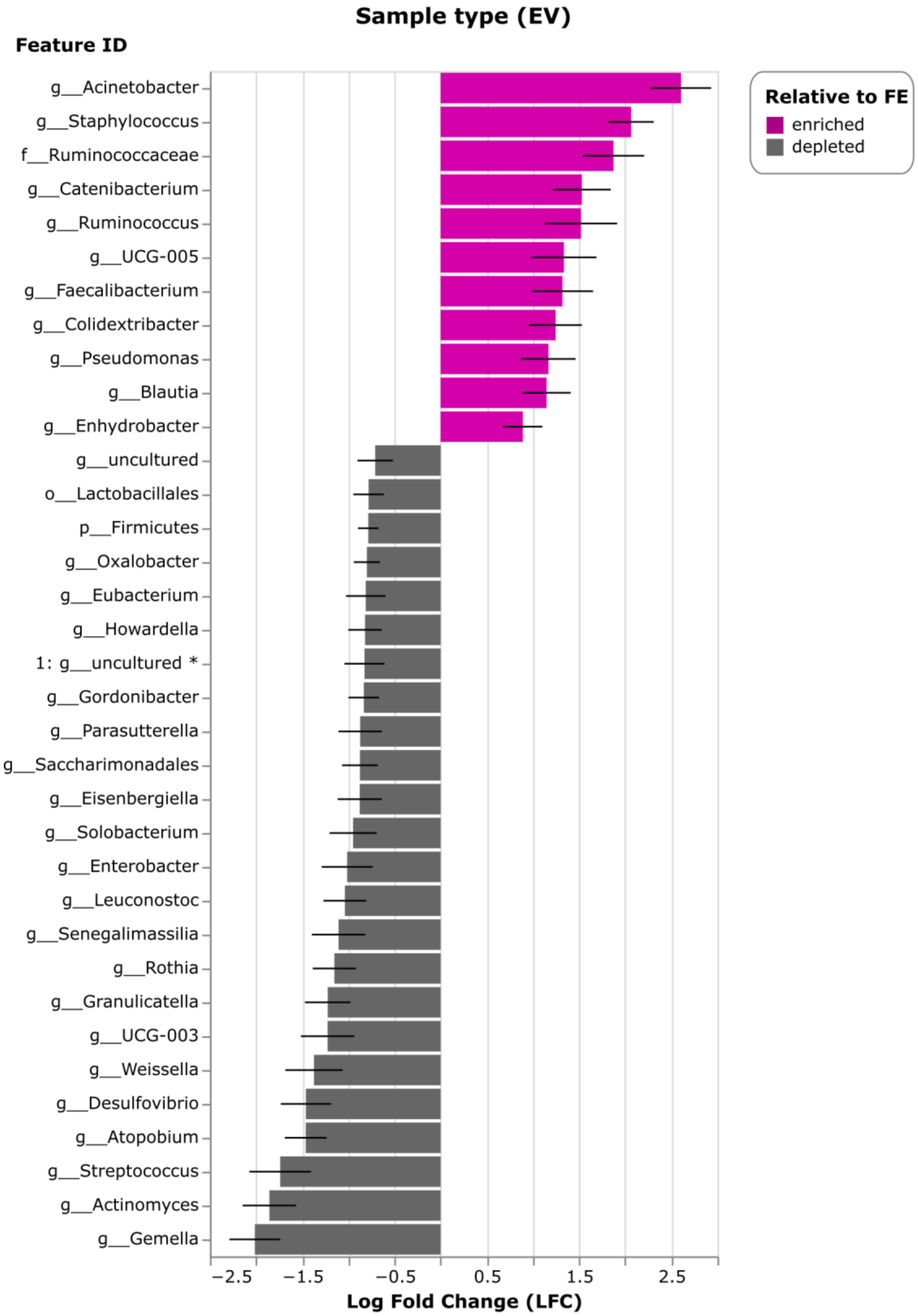

Supplemental figure 11. Differentially abundant genera between gut microbiota-derived EV and fecal samples in the colorectal cancer dataset, with 95% confidence intervals for the mean differential abundance for all genera. Genera with positive LFC values (pink) are enriched, and genera with negative LFC values (grey) are depleted in the gut microbiota-derived EVs compared to the fecal samples.
