# Supplementary material for: Gut microbiota-derived extracellular vesicles form a distinct entity from gut microbiota
Source: mSystems. 2025 Apr 29;10(5):e00311-25. doi: 10.1128/msystems.00311-25 (PMC12090791; doi:10.1128/msystems.00311-25)
Supplement: Supplemental figures, part 2 — Figures S12 to S20. [file msystems.00311-25-s0002.pdf]

Supplemental figures (12-20)

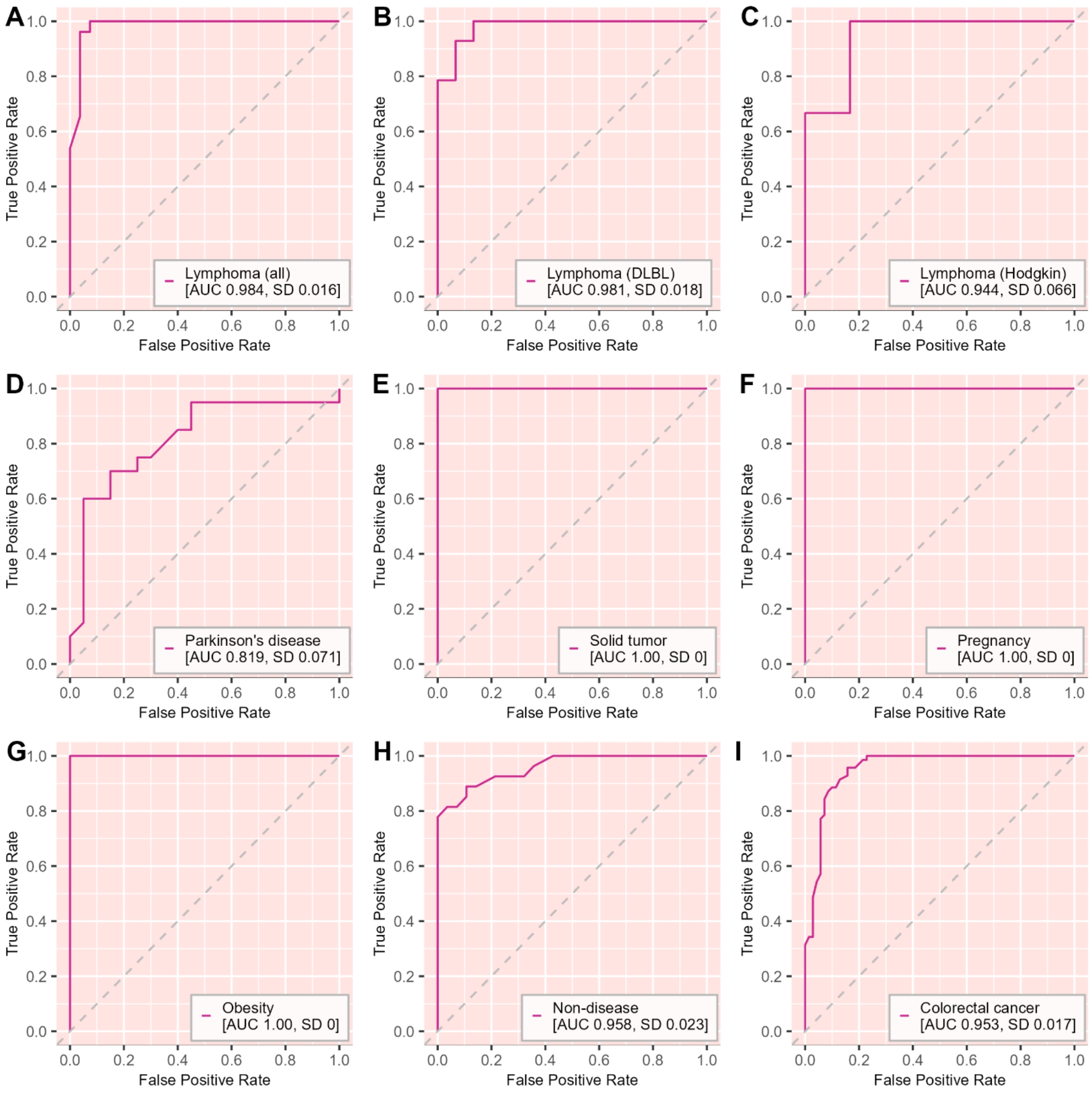

Supplemental figure 12. Machine learning classification of gut microbiota and paired gut microbiota-derived EV samples within each dataset using random forest classifier. A) Lymphoma (n = 53, n_{EV} = 27, n_{feces} = 26); B) Lymphoma, diffuse large B cell lymphoma (n =29, n_{EV} = 15, n_{feces} = 14); C) Hodgkin's lymphoma (n = 12, n_{EV} =6, n_{feces} =6); D) Parkinson's disease (n = 40, n_{EV} = 20, n_{feces} = 20); E) Solid tumor (n = 52, n_{EV} = 25, n_{feces} = 27); F) Pregnancy (n = 45, n_{EV} = 22, n_{feces} = 23); G) Obesity (n = 59, n_{EV} = 29, n_{feces} = 30); H) Non-disease (n = 55, n_{EV} = 28, n_{feces} = 27); I) Colorectal cancer (n = 140, n_{EV} = 70, n_{feces} = 70). The classification accuracy is presented as area under the curve (AUC) of receiver operating characteristic (ROC) figures. Standard deviation (SD) for AUC values are indicated in each dataset. Dashed lines indicate random classification performance.

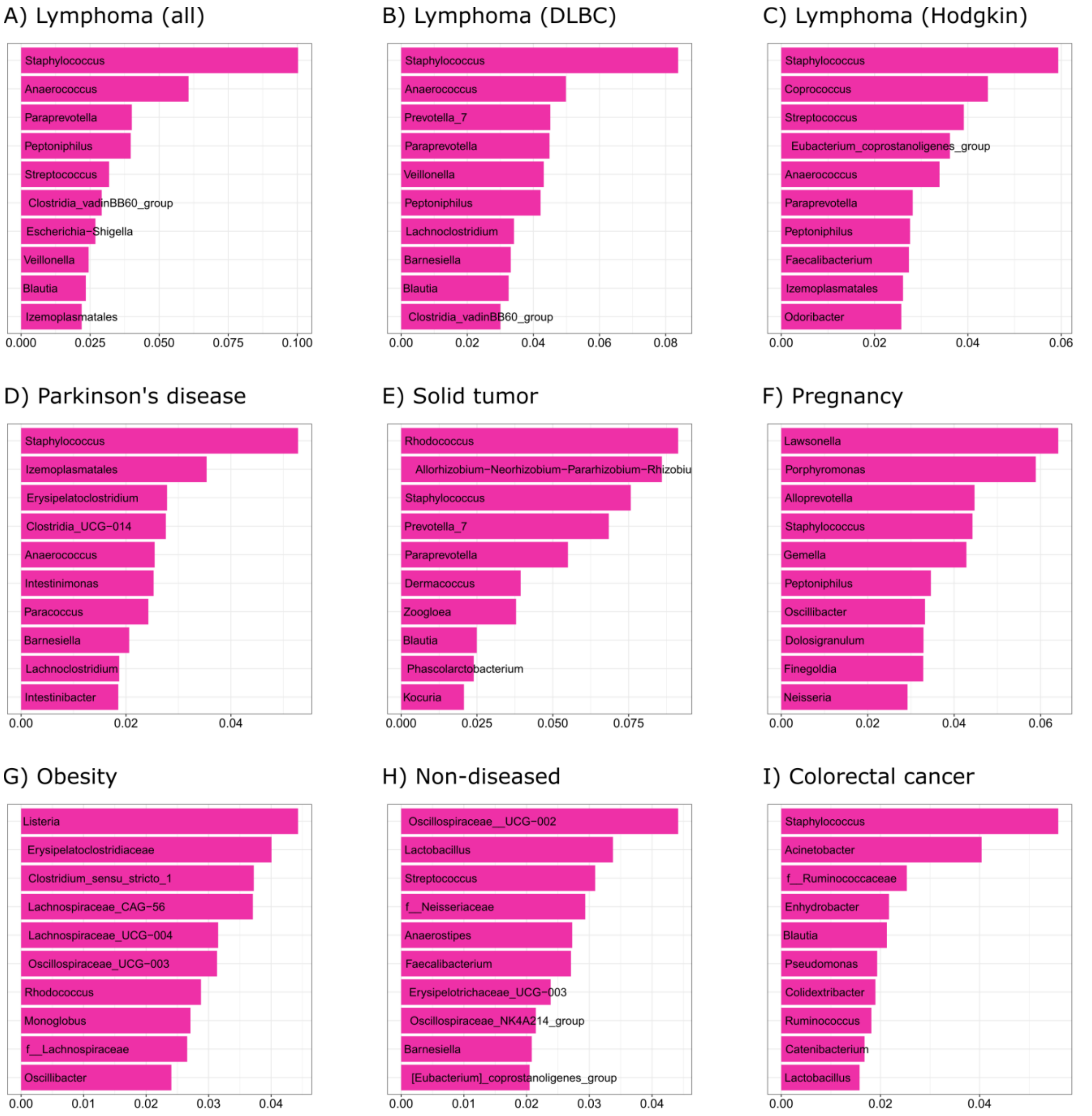

Supplemental figure 13. The most important taxonomic features for random forest classification of gut microbiota and gut microbiota-derived EV samples in each dataset. Feature importances are presented as importance scores for each dataset. *Allorhizobium-Neorhizobium-Pararhizobium-Rhizobium.

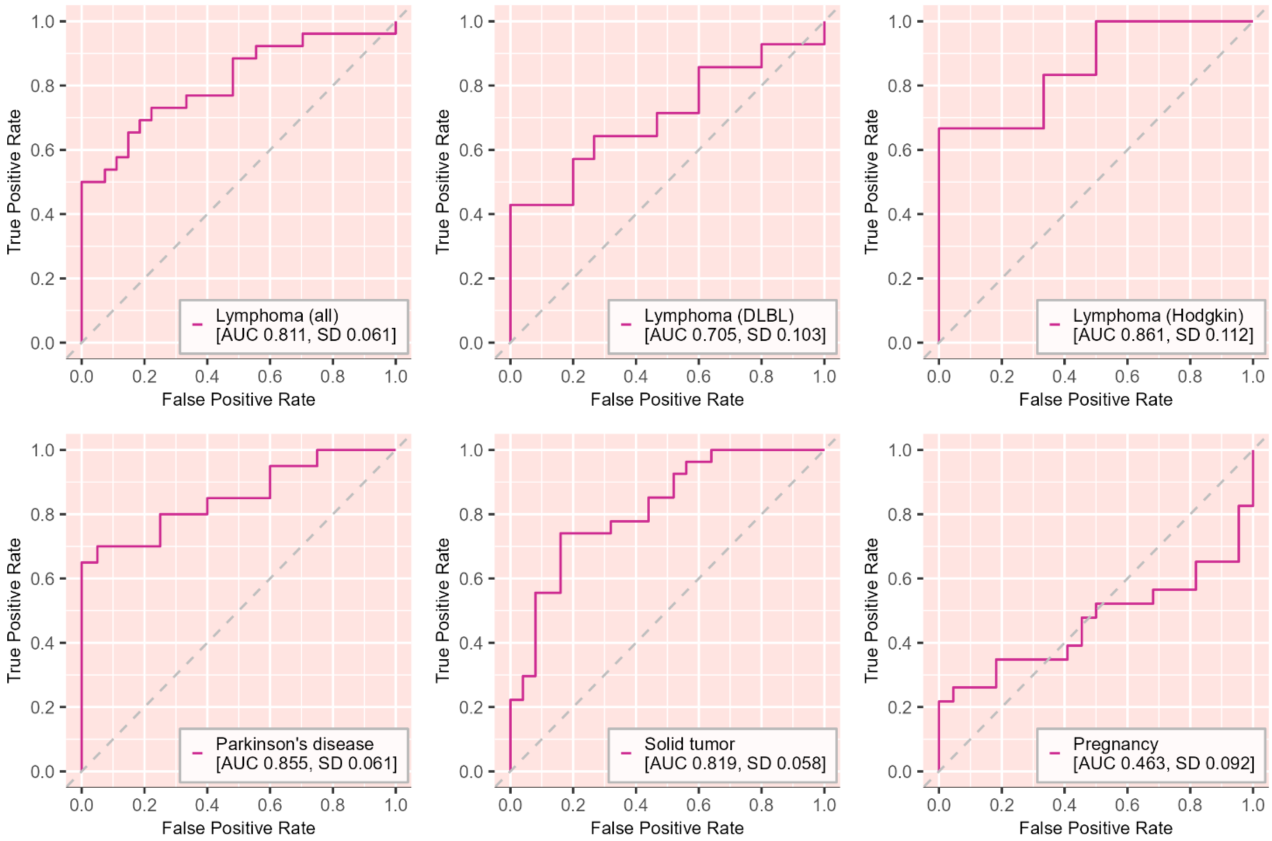

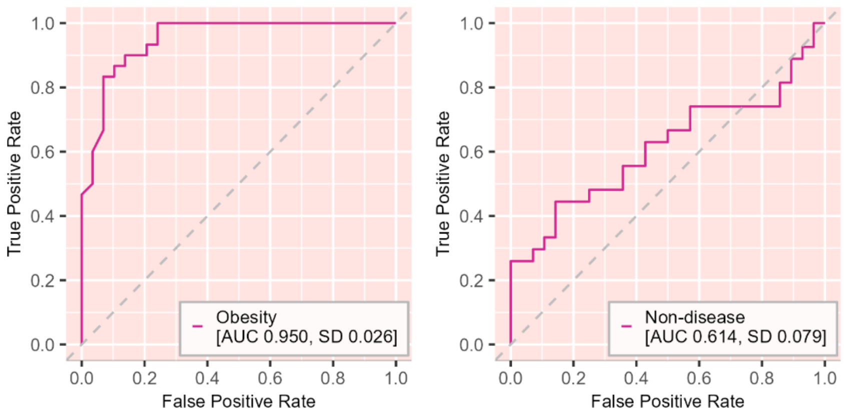

Dataset	AUC-value
Lymphoma (all)	0.811
Lymphoma (DLBL)	0.705
Lymphoma (Hodgkin)	0.861
Parkinson's disease	0.855
Solid tumor	0.819
Pregnancy	0.463
Obesity	0.950
Non-disease	0.614
Colorectal cancer	Training dataset

Supplemental figure 14. Cross-study machine learning classification of gut microbiota and paired gut microbiota-derived EV samples using random forest classifier. A) Lymphoma ($n = 53$, $n_{EV} = 27$, $n_{feces} = 26$); B) Lymphoma, diffuse large B cell lymphoma ($n =29$, $n_{EV} = 15$, $n_{feces} = 14$); C) Hodgkin's lymphoma ($n = 12$, $n_{EV} =6$, $n_{feces} =6$); D) Parkinson's disease ($n = 40$, $n_{EV} = 20$, $n_{feces} = 20$); E) Solid tumor ($n = 52$, $n_{EV} = 25$, $n_{feces} = 27$); F) Pregnancy ($n = 45$, $n_{EV} = 22$, $n_{feces} = 23$); G) Obesity ($n = 59$, $n_{EV} = 29$, $n_{feces} = 30$); H) Non-disease ($n = 55$, $n_{EV} = 28$, $n_{feces} = 27$); I) Colorectal cancer ($n = 140$, $n_{EV} = 70$, $n_{feces} = 70$). The classification accuracy is presented as area under the curve (AUC) of receiver operating characteristic (ROC) figures. Standard deviation (SD) for AUC values is indicated in each dataset. Dashed lines indicate random classification performance.

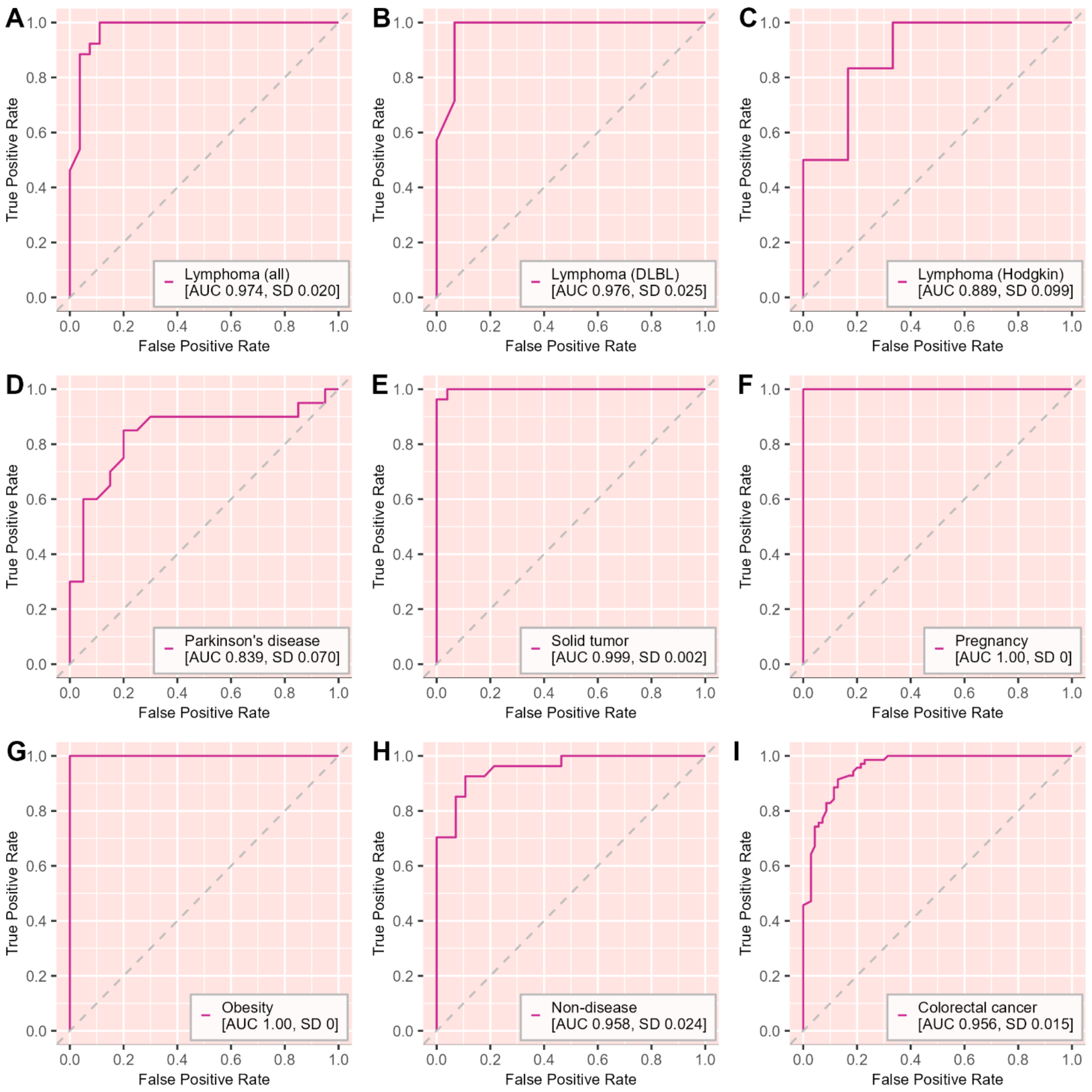

Supplemental figure 15. Machine learning classification of gut microbiota and paired gut microbiota-derived EV samples within each dataset using extra trees classifier. A) Lymphoma (n = 53, n_{EV} = 27, n_{feces} = 26); B) Lymphoma, diffuse large B cell lymphoma (n =29, n_{EV} = 15, n_{feces} = 14); C) Hodgkin's lymphoma (n = 12, n_{EV} =6, n_{feces} =6); D) Parkinson's disease (n = 40, n_{EV} = 20, n_{feces} = 20); E) Solid tumor (n = 52, n_{EV} = 25, n_{feces} = 27); F) Pregnancy (n = 45, n_{EV} = 22, n_{feces} = 23); G) Obesity (n = 59, n_{EV} = 29, n_{feces} = 30); H) Non-disease (n = 55, n_{EV} = 28, n_{feces} = 27); I) Colorectal cancer (n = 140, n_{EV} = 70, n_{feces} = 70). The classification accuracy is presented as area under the curve (AUC) of receiver operating characteristic (ROC) figures. Standard deviation (SD) for AUC values is indicated in each dataset. Dashed lines indicate random classification performance.

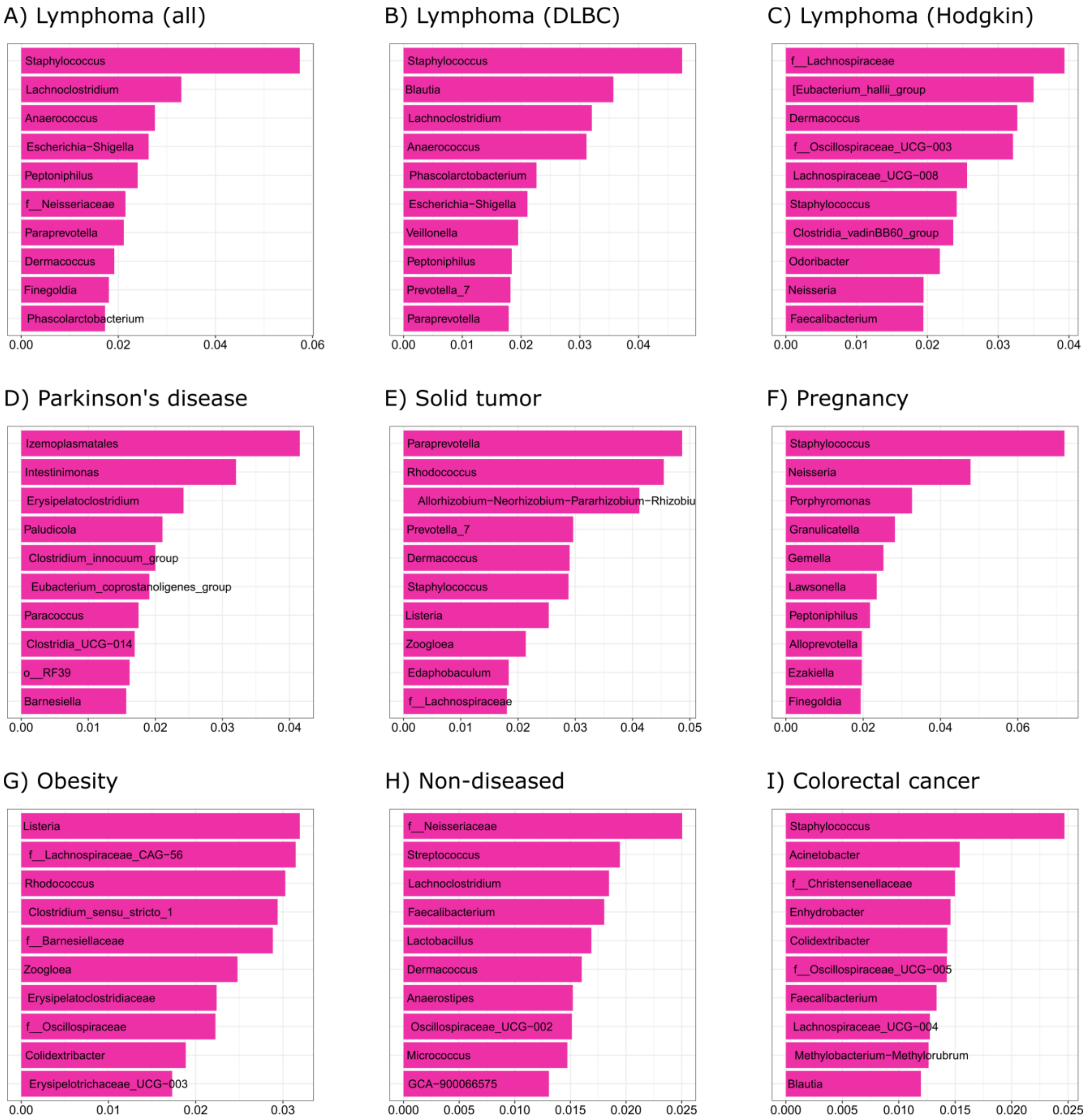

Supplemental figure 16. The most important taxonomic features for extra trees classification of gut microbiota and gut microbiota-derived EV samples in each dataset. Feature importances are presented as importance scores for each dataset. *Allorhizobium-Neorhizobium-Pararhizobium-Rhizobium.

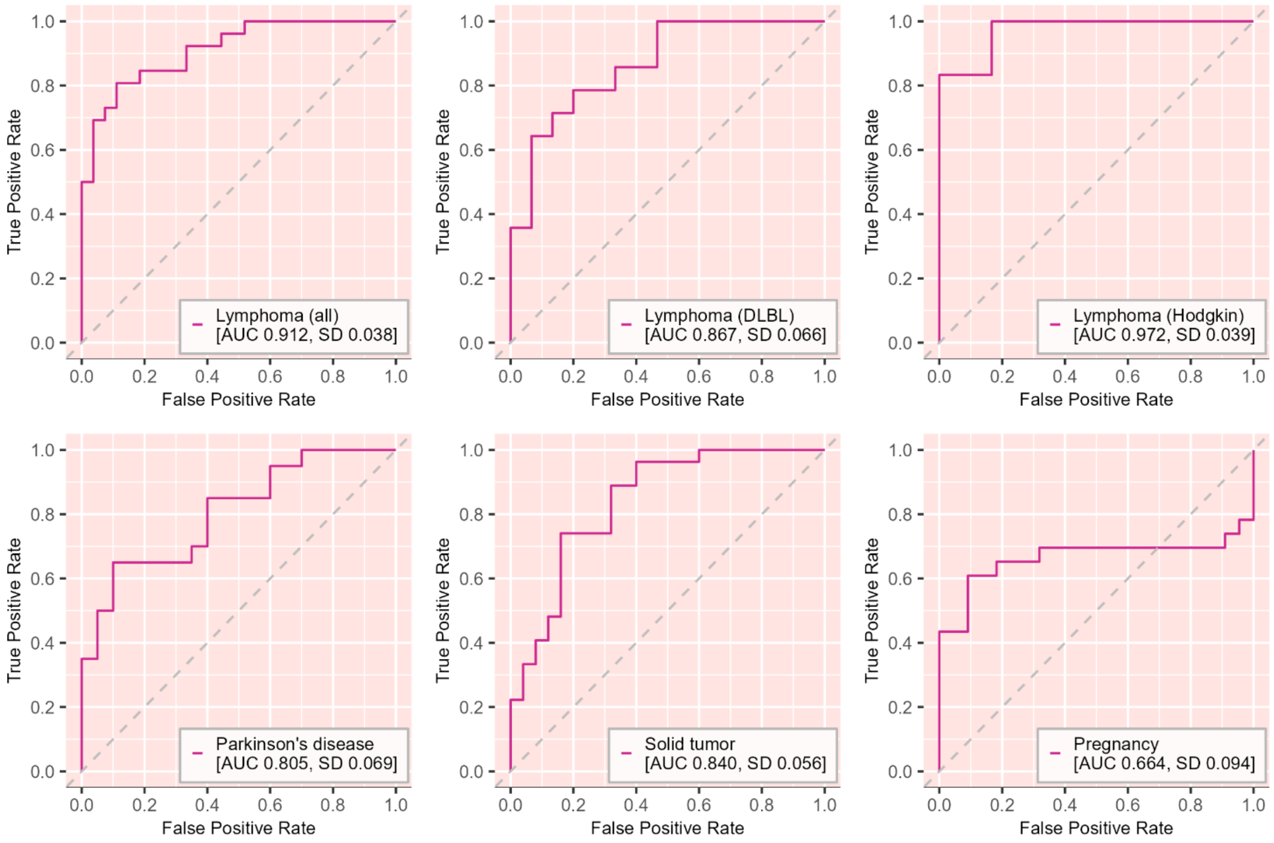

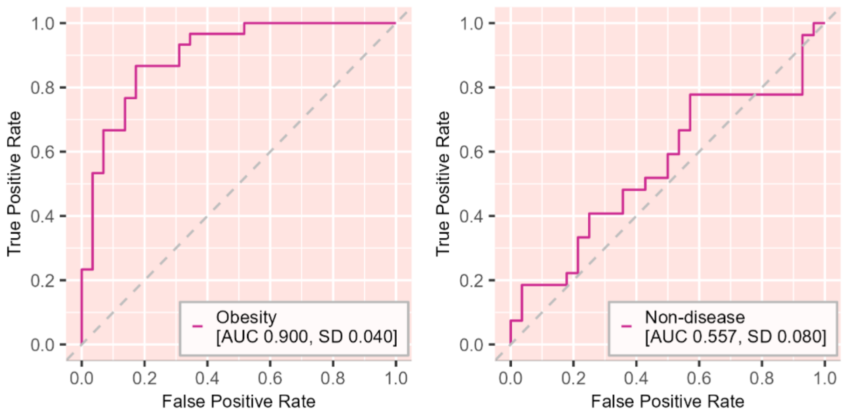

Dataset	AUC-value
Lymphoma (all)	0.912
Lymphoma (DLBL)	0.867
Lymphoma (Hodgkin)	0.972
Parkinson's disease	0.805
Solid tumor	0.840
Pregnancy	0.664
Obesity	0.900
Non-disease	0.557
Colorectal cancer	Training dataset

Supplemental figure 17. Cross-study machine learning classification of gut microbiota and paired gut microbiota-derived EV samples using extra trees classifier. A) Lymphoma ($n = 53$, $n_{EV} = 27$, $n_{feces} = 26$); B) Lymphoma, diffuse large B cell lymphoma ($n = 29$, $n_{EV} = 15$, $n_{feces} = 14$); C) Hodgkin's lymphoma ($n = 12$, $n_{EV} = 6$, $n_{feces} = 6$); D) Parkinson's disease ($n = 40$, $n_{EV} = 20$, $n_{feces} = 20$); E) Solid tumor ($n = 52$, $n_{EV} = 25$, $n_{feces} = 27$); F) Pregnancy ($n = 45$, $n_{EV} = 22$, $n_{feces} = 23$); G) Obesity ($n = 59$, $n_{EV} = 29$, $n_{feces} = 30$); H) Non-disease ($n = 55$, $n_{EV} = 28$, $n_{feces} = 27$); I) Colorectal cancer ($n = 140$, $n_{EV} = 70$, $n_{feces} = 70$). The classification accuracy is presented as area under the curve (AUC) of receiver operating characteristic (ROC) figures. Standard deviation (SD) for AUC values is indicated in each dataset. Dashed lines indicate random classification performance.

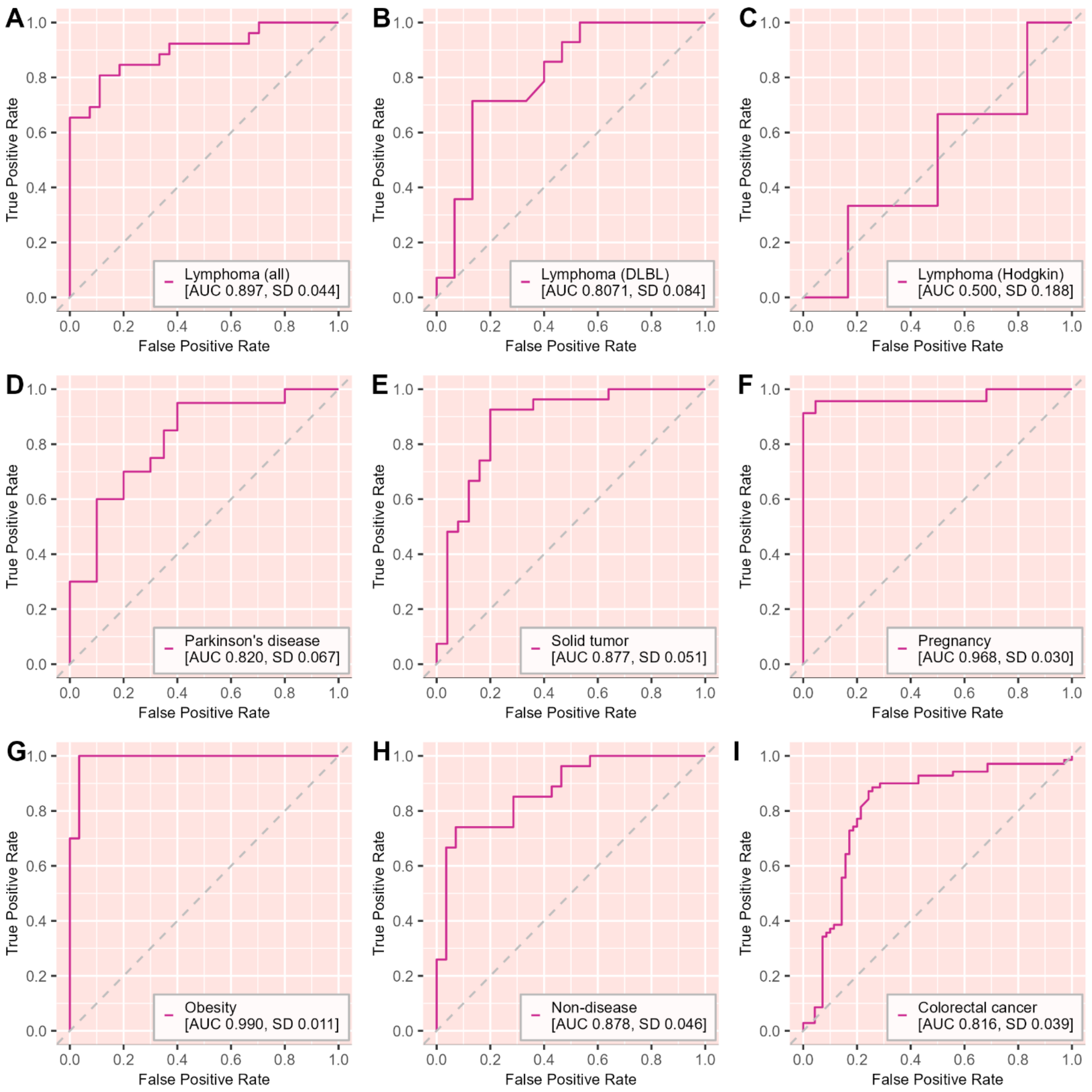

Supplemental figure 18. Machine learning classification of gut microbiota and paired gut microbiota-derived EV samples within each dataset using linear support vector classification classifier. A) Lymphoma (n = 53, n_{EV} = 27, n_{feces} = 26); B) Lymphoma, diffuse large B cell lymphoma (n =29, n_{EV} = 15, n_{feces} = 14); C) Hodgkin's lymphoma (n = 12, n_{EV} =6, n_{feces} =6); D) Parkinson's disease (n = 40, n_{EV} = 20, n_{feces} = 20); E) Solid tumor (n = 52, n_{EV} = 25, n_{feces} = 27); F) Pregnancy (n = 45, n_{EV} = 22, n_{feces} = 23); G) Obesity (n = 59, n_{EV} = 29, n_{feces} = 30); H) Non-disease (n = 55, n_{EV} = 28, n_{feces} = 27); I) Colorectal cancer (n = 140, n_{EV} = 70, n_{feces} = 70). The classification accuracy is presented as area under the curve (AUC) of receiver operating characteristic (ROC) figures. Standard deviation (SD) for AUC values is indicated in each dataset. Dashed lines indicate random classification performance.

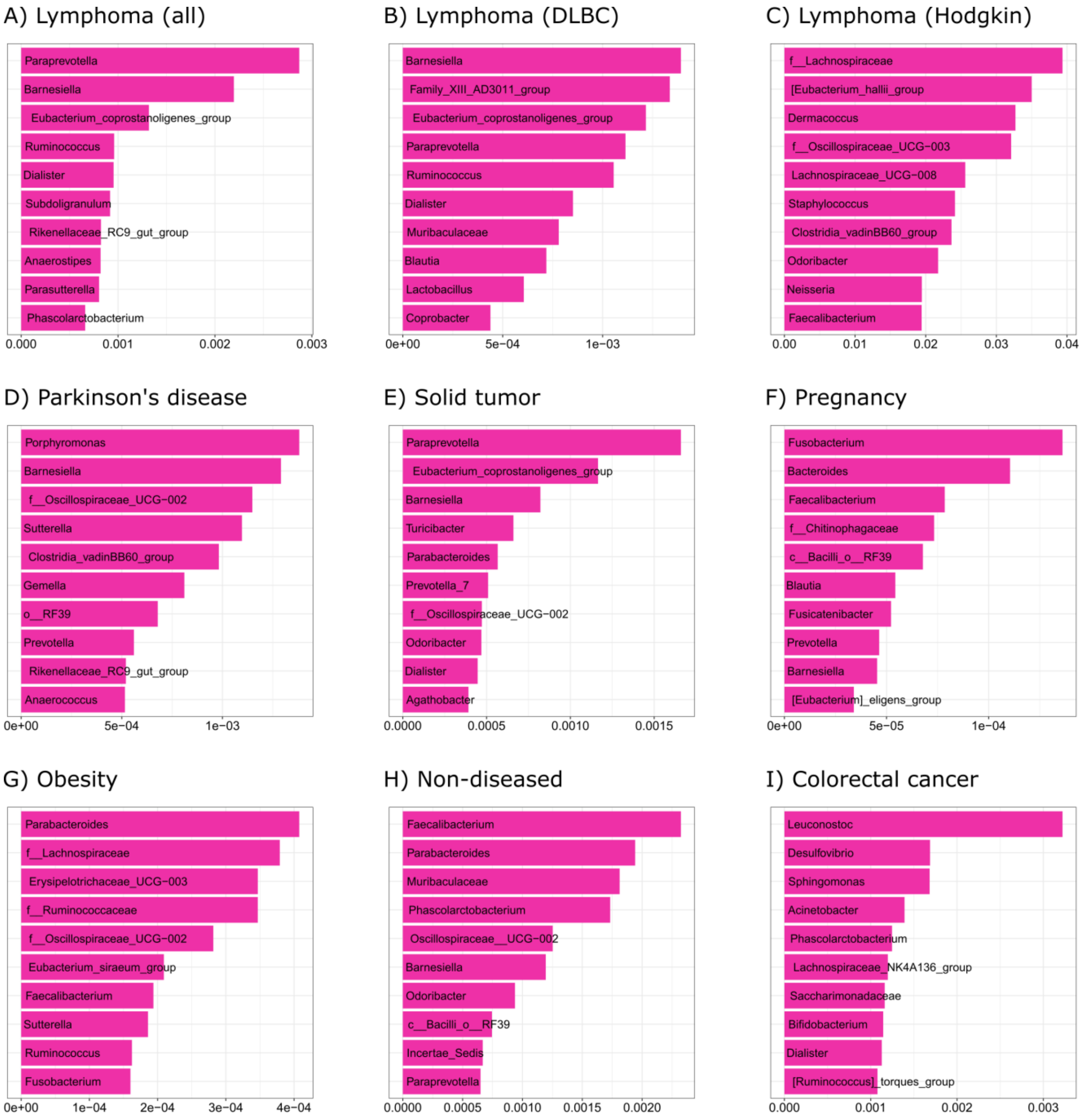

Supplemental figure 19. The most important taxonomic features for linear support vector classifier classification of gut microbiota and gut microbiota-derived EV samples in each dataset. Feature importances are presented as importance scores for each dataset.

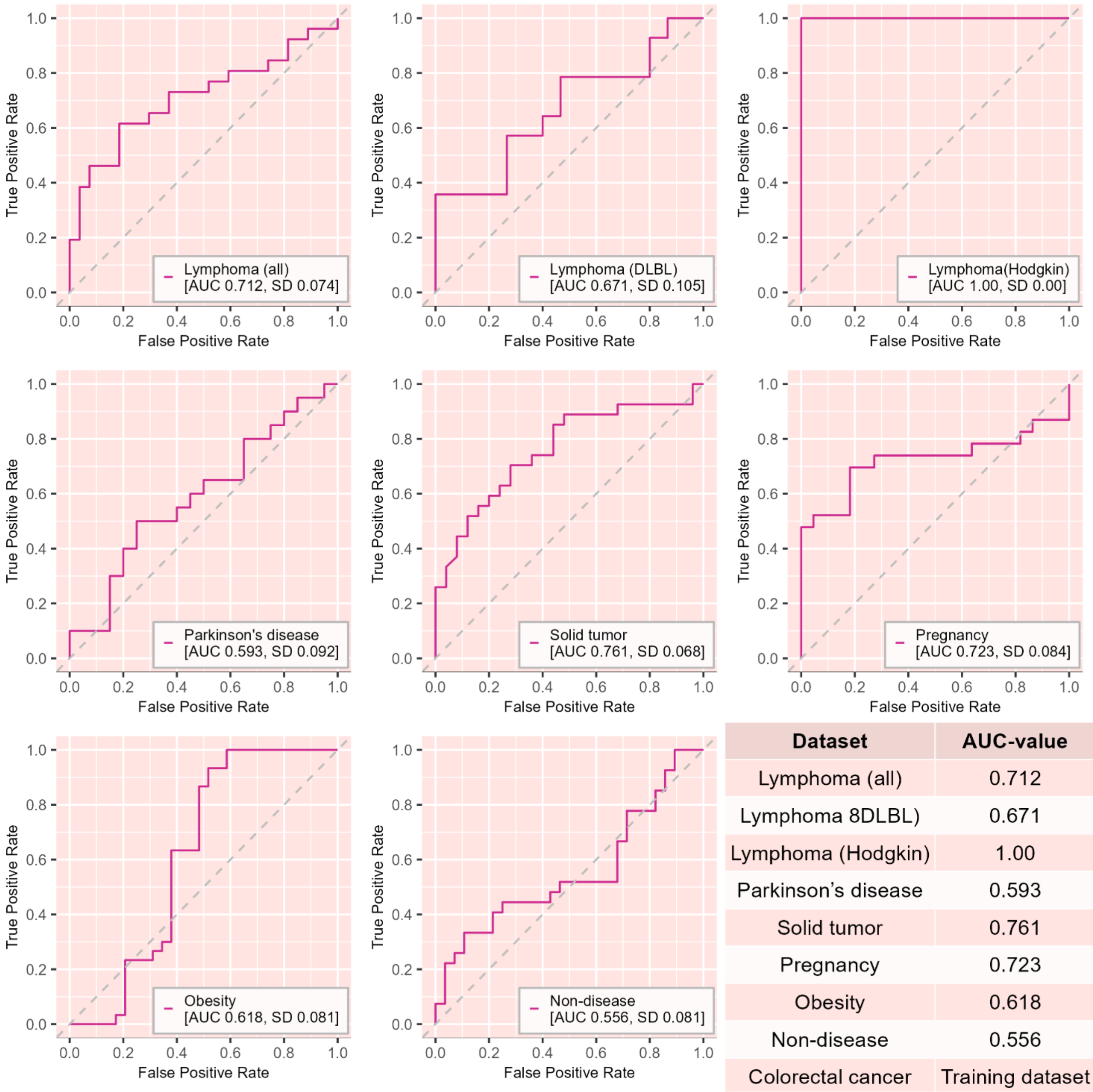

Dataset	AUC-value
Lymphoma (all)	0.712
Lymphoma 8DLBL)	0.671
Lymphoma (Hodgkin)	1.00
Parkinson's disease	0.593
Solid tumor	0.761
Pregnancy	0.723
Obesity	0.618
Non-disease	0.556
Colorectal cancer	Training dataset

Supplemental figure 20. Cross-study machine learning classification of gut microbiota and paired gut microbiota-derived EV samples using linear support vector classification classifier. A) Lymphoma (n = 53, n_{EV} = 27, n_{feces} = 26); B) Lymphoma, diffuse large B cell lymphoma (n =29, n_{EV} = 15, n_{feces} = 14); C) Hodgkin's lymphoma (n = 12, n_{EV} =6, n_{feces} =6); D) Parkinson's disease (n = 40, n_{EV} = 20, n_{feces} = 20); E) Solid tumor (n = 52, n_{EV} = 25, n_{feces} = 27); F) Pregnancy (n = 45, n_{EV} = 22, n_{feces} = 23); G) Obesity (n = 59, n_{EV} = 29, n_{feces} = 30); H) Non-disease (n = 55, n_{EV} = 28, n_{feces} = 27); I) Colorectal cancer (n = 140, n_{EV} = 70, n_{feces} = 70). The classification accuracy is presented as area under the curve (AUC) of receiver operating characteristic (ROC) figures. Standard deviation (SD) for AUC values is indicated in each dataset. Dashed lines indicate random classification performance.
