# Supplementary material for: Gut microbiota-derived extracellular vesicles form a distinct entity from gut microbiota
Source: mSystems. 2025 Apr 29;10(5):e00311-25. doi: 10.1128/msystems.00311-25 (PMC12090791; doi:10.1128/msystems.00311-25)
Supplement: Supplemental tables — Tables S1 to S21. [file msystems.00311-25-s0003.pdf]

Supplemental tables

Supplemental table 1. Additional clinical data of the clinical cohorts.

Clinical cohort	n	
Lymphoma	28	Patients with Diffuse large B-cell lymphoma, n = 16<br>Patients with Hodgkin lymphoma, n = 6<br>Patients with Mantle cell lymphoma, n = 3<br>Patients with Mature T/NK-cell lymphoma, n = 1<br>Patients with other types of lymphoma (not specified), n = 2
Solid tumor	28	Patients with non-small cell lung cancer, n = 15<br>Patients with malignant melanoma, n = 5<br>Patients with renal carcinoma, n = 5<br>Patients with urothelial carcinoma, n = 1<br>Patients with head and neck squamocellular carcinoma, n = 1<br>Patients with cancer of unknown origin, n = 1
Pregnancy	23	Full-term pregnancies, n = 23*<br>*samples collected before or after cesarean delivery
Obesity	30	BMI, mean (SD) = 44.5 (5.7)<br>Patients with type 2 diabetes, n = 16<br>Patients without type 2 diabetes, n = 14

Supplemental table 2. Search queries used to identify published 16S rRNA gene sequencing datasets.

Database	Date	Search query
PubMed	September 1st 2023	(((((“High-Throughput Nucleotide Sequencing”[MeSH]) OR (“next generation sequencing”[Text Word])) OR (“NGS”[Text Word])) OR (rna, 16s ribosomal[MeSH Terms] OR (16S*[Text Word]) OR (metagenome[MeSH Terms]) OR (metagenom*[Text Word]) OR (composition[Title/Abstract]) OR (abundance[Title/Abstract]))) AND (“Microbiota”[MeSH] OR “microbiota”[Text Word] OR “microbiome”[Text Word])) AND (“Extracellular Vesicles”[MeSH] OR “outer membrane vesicle*”[Text Word] OR “extracellular vesicle*”[Text Word])
Scopus	September 1st 2023	: (TITLE-ABS-KEY (“*microbiota*” OR “*microbiome*”)) AND (TITLE-ABS-KEY (“*extracellular vesicle*” OR “*outer membrane vesicle*”)) AND (TITLE-ABS-KEY (“16S*” OR “metagenom*” OR “composition” OR “abundance” OR “next generation sequencing” OR “NGS”))
Web of Science	September 1st 2023	((AB=(“microbiota*” OR “microbiome*”)) AND AB=(“extracellular vesicle*” OR “outer membrane vesicle*”)) AND AB=(“16S*” OR “metagenom*” OR “composition” OR “abundance” OR “next generation sequencing” OR “NGS”)

Supplemental table 3. Relative abundances as percentages of genera present in gut microbiota (FE, left) and genera secreting gut microbiota-derived EVs (EV, right) in the lymphoma dataset. The 20 most abundant genera have been listed, and the less abundant genera have been combined in the "other" category.

Genus	FE (%)	Genus	EV (%)
Bacteroides	34.50	Bacteroides	31.74
Faecalibacterium	7.53	Alistipes	9.81
Prevotella_9	6.45	Prevotella_9	8.89
Alistipes	5.40	Faecalibacterium	4.89
Parabacteroides	3.30	Staphylococcus	3.02
Erysipelotrichaceae_UCG-003	3.11	Odoribacter	2.95
Barnesiella	2.68	Parabacteroides	2.74
Ruminococcus	1.98	Christensenellaceae_R-7_group	2.08
Christensenellaceae_R-7_group	1.93	Erysipelotrichaceae_UCG-003	2.08
Prevotellaceae_UCG-001	1.87	Izemoplasmatales	2.06
Blautia	1.84	Streptococcus	1.89
Paraprevotella	1.80	Roseburia	1.49
Prevotellaceae_NK3B31_group	1.73	Sutterella	1.41
Sutterella	1.53	Prevotellaceae_NK3B31_group	1.29
Agathobacter	1.51	Anaerococcus	1.07
Eubacterium_coprostanoligenes_group	1.41	Lachnospiraceae_NK4A136_group	1.00
Roseburia	1.37	Peptoniphilus	0.99
Lactobacillus	1.37	Subdoligranulum	0.98
Subdoligranulum	1.28	Ruminococcus	0.92
Lachnospiraceae_NK4A136_group	1.24	Prevotellaceae_UCG-001	0.90
other	16.18	other	17.79

Supplemental table 4. Relative abundances as percentages of genera present in gut microbiota (FE, left) and genera secreting gut microbiota-derived EVs (EV, right) in the Diffuse large B cell lymphoma subset of the lymphoma dataset. The 20 most abundant genera have been listed, and the less abundant genera have been combined in the “other” category.

Genus	FE (%)	Genus	EV (%)
Bacteroides	38.87	Bacteroides	36.10
Faecalibacterium	7.79	Alistipes	8.61
Alistipes	5.30	Faecalibacterium	5.59
Erysipelotrichaceae_UCG-003	4.01	Staphylococcus	3.53
Prevotellaceae_UCG-001	2.98	Prevotella_9	3.39
Barnesiella	2.55	Parabacteroides	2.72
Parabacteroides	2.42	Christensenellaceae_R-7_group	2.42
Ruminococcus	2.31	Erysipelotrichaceae_UCG-003	2.30
Blautia	2.17	Streptococcus	2.11
Agathobacter	1.93	Izemoplasmatales	1.91
Sutterella	1.65	Prevotellaceae_UCG-001	1.77
Prevotella_9	1.45	Odoribacter	1.57
Eubacterium_coprostanoligenes_group	1.45	Sutterella	1.48
Subdoligranulum	1.42	Roseburia	1.29
Prevotellaceae_NK3B31_group	1.41	Lactobacillus	1.17
Roseburia	1.37	Ruminococcus	1.08
Paraprevotella	1.34	Prevotellaceae_NK3B31_group	0.97
Lachnospiraceae_NK4A136_group	1.28	Lachnospiraceae_NK4A136_group	0.94
Muribaculaceae	0.98	Blautia	0.90
Lactobacillus	0.95	o__Rhodospirillales	0.87
other	16.35	other	19.27

Supplemental table 5. Relative abundances as percentages of genera present in gut microbiota (FE, left) and genera secreting gut microbiota-derived EVs (EV, right) in the Hodgkin lymphoma subset of the lymphoma dataset. The 20 most abundant genera have been listed, and the less abundant genera have been combined in the “other” category.

Genus	FE (%)	Genus	EV (%)
Bacteroides	30.20	Bacteroides	30.67
Prevotella_9	12.81	Prevotella_9	15.10
Faecalibacterium	8.72	Alistipes	14.74
Alistipes	6.03	Odoribacter	4.98
Parabacteroides	5.05	Prevotellaceae_NK3B31_group	2.88
Prevotellaceae_NK3B31_group	3.81	Faecalibacterium	2.79
Barnesiella	3.61	Staphylococcus	2.38
Lactobacillus	3.42	Peptoniphilus	2.07
Paraprevotella	3.15	Izemoplasmatales	2.01
Erysipelotrichaceae_UCG-003	1.76	Erysipelatoclostridium	1.88
Roseburia	1.60	Parabacteroides	1.81
c__Bacilli_RF39	1.38	Streptococcus	1.63
Subdoligranulum	1.28	Anaerococcus	1.61
Ruminococcus	1.18	Barnesiella	1.39
Sutterella	1.17	Roseburia	1.28
Clostridia_UCG-014	1.12	Subdoligranulum	1.23
Phascolarctobacterium	1.04	Sutterella	1.08
Blautia	0.91	Bacillus	0.50
Odoribacter	0.89	Blautia	0.50
Agathobacter	0.86	Ruminococcus	0.42
other	10.03	other	9.05

Supplemental table 6. Relative abundances as percentages of genera present in gut microbiota (FE, left) and genera secreting gut microbiota -derived EVs (EV, right) in the Parkinson's disease dataset. The 20 most abundant genera have been listed, and the less abundant genera have been combined in the "other" category.

Genus	FE (%)	Genus	EV (%)
Bacteroides	27.54	Bacteroides	21.58
Alistipes	10.88	Izemoplasmatales	19.24
Christensenellaceae_R-7_group	4.30	Alistipes	15.70
Rikenellaceae_RC9_gut_group	3.93	Christensenellaceae_R-7_group	3.95
Streptococcus	3.73	f__Ruminococcaceae	3.57
Prevotella_9	3.54	Prevotella_9	3.25
Parabacteroides	3.13	Clostridia_vadinBB60_group	3.09
Izemoplasmatales	3.12	Streptococcus	2.59
Faecalibacterium	2.83	Staphylococcus	2.58
c__Bacilli_TF39	2.76	Faecalibacterium	2.00
Clostridia_vadinBB60_group	2.66	Parabacteroides	1.59
Clostridia_UCG-014	2.38	Lactobacillus	1.38
Oscillospiraceae_UCG-002	1.56	UCG-005	1.30
Subdoligranulum	1.52	Odoribacter	1.08
Staphylococcus	1.44	Sutterella	0.91
Eubacterium_coprostanoligenes_group	1.28	Subdoligranulum	0.77
f__Ruminococcaceae	1.21	Oscillospirales_UCG-010	0.75
Sutterella	1.14	Erysipelotrichaceae_UCG-003	0.74
Barnesiella	1.07	Agathobacter	0.55
Odoribacter	1.06	Blautia	0.49
other	18.91	other	12.88

Supplemental table 7. Relative abundances as percentages of genera present in gut microbiota (FE, left) and genera secreting gut microbiota -derived EVs (EV, right) in the solid tumor dataset. The 20 most abundant genera have been listed, and the less abundant genera have been combined in the "other" category.

Genus	FE (%)	Genus	EV (%)
Bacteroides	38.78	Bacteroides	29.28
Alistipes	10.21	Alistipes	14.77
Faecalibacterium	4.55	Prevotella_9	5.77
Paraprevotella	2.85	Rhodococcus	5.66
Parabacteroides	2.60	Izemoplasmatales	4.13
Blautia	2.08	Faecalibacterium	2.73
Sutterella	2.02	Sutterella	2.26
Ruminococcus	2.01	Eubacterium_siraeum_group	1.94
Agathobacter	1.72	Roseburia	1.76
c__Bacilli_RF39	1.70	Christensenellaceae_R-7_group	1.73
Streptococcus	1.69	Parabacteroides	1.61
Eubacterium_coprostanoligenes_group	1.55	Ruminococcaceae_CAG-352	1.39
Prevotella_9	1.48	Staphylococcus	1.02
Roseburia	1.41	Clostridia_vadinBB60_group	0.98
Erysipelotrichaceae_UCG-003	1.32	Odoribacter	0.96
Barnesiella	1.28	Streptococcus	0.96
Erysipelatoclostridium	1.23	Lactobacillus	0.94
Dialister	1.19	Agathobacter	0.92
Prevotella_7	1.15	f__Ruminococcaceae	0.89
Odoribacter	1.05	Lachnospiraceae_NK4A136_group	0.88
other	18.14	other	19.42

Supplemental table 8. Relative abundances as percentages of genera present in gut microbiota (FE, left) and genera secreting gut microbiota -derived EVs (EV, right) in the pregnancy dataset. The 20 most abundant genera have been listed, and the less abundant genera have been combined in the "other" category.

Genus	FE (%)	Genus	EV (%)
Bacteroides	30.40	Staphylococcus	30.81
Alistipes	11.91	Streptococcus	16.99
Oscillospiraceae_UCG-002	4.01	Anaerococcus	7.30
Prevotella	3.21	Alloprevotella	4.60
Faecalibacterium	2.90	Porphyromonas	3.05
f__Ruminococcaceae	2.79	Bacteroides	2.84
Turicibacter	2.10	Gemella	2.63
Blautia	2.03	Prevotella	2.16
Erysipelotrichaceae_UCG-003	1.98	Lawsonella	2.07
Clostridia_UCG-014	1.85	Peptoniphilus	1.71
Barnesiella	1.83	Williamsia	1.66
Agathobacter	1.55	Micrococcus	1.47
Fusicatenibacter	1.52	Neisseria	1.39
c__Bacilli_RF39	1.45	Deinococcus	1.32
Eubacterium_coprostanoligenes_group	1.42	Granulicatella	1.31
Trichococcus	1.26	Finegoldia	1.16
Parabacteroides	1.06	Lactobacillus	0.94
Odoribacter	1.06	Methylobacterium-Methylorubrum	0.67
f__Lachnospiraceae	1.01	Fusobacterium	0.63
Subdoligranulum	1.00	Veillonella	0.62
other	23.66	other	14.67

Supplemental table 9. Relative abundances as percentages of genera present in gut microbiota (FE, left) and genera secreting gut microbiota -derived EVs (EV, right) in the obesity dataset. The 20 most abundant genera have been listed, and the less abundant genera have been combined in the "other" category.

Genus	FE (%)	Genus	EV (%)
Bacteroides	30.15	Bacteroides	23.67
Alistipes	9.80	Staphylococcus	17.23
Prevotella	6.15	Streptococcus	9.83
Faecalibacterium	5.71	Alistipes	5.37
Parabacteroides	3.41	Anaerococcus	3.38
f__Lachnospiraceae	1.95	Lactobacillus	2.12
Erysipelotrichaceae_UCG-003	1.88	Prevotella	1.86
Roseburia	1.86	f__Rhodobacteraceae	1.51
Ruminococcus	1.84	Faecalibacterium	1.48
Agathobacter	1.83	Finegoldia	1.37
Blautia	1.82	Christensenellaceae_R-7_group	1.29
Eubacterium_siraeum_group	1.67	Gemella	1.20
Sutterella	1.57	Agathobacter	1.14
f__Ruminococcaceae	1.52	Blautia	1.07
Oscillospiraceae_UCG-002	1.49	Neisseria	0.98
Eubacterium_coprostanoligenes_group	1.45	Peptoniphilus	0.89
Christensenellaceae_R-7_group	1.44	Odoribacter	0.86
Staphylococcus	1.32	Izemoplasmatales	0.80
Subdoligranulum	1.19	Sutterella	0.77
Odoribacter	1.14	Parabacteroides	0.73
other	20.83	other	22.45

Supplemental table 10. Relative abundances as percentages of genera present in gut microbiota (FE, left) and genera secreting gut microbiota -derived EVs (EV, right) in the non-diseased dataset. The 20 most abundant genera have been listed, and the less abundant genera have been combined in the "other" category.

Genus	FE (%)	Genus	EV (%)
Bacteroides	26.04	Bacteroides	30.21
Alistipes	6.50	Prevotella	8.33
Faecalibacterium	5.61	Staphylococcus	6.22
Prevotella	5.09	Alistipes	5.71
Sutterella	3.30	Sutterella	4.87
Barnesiella	2.38	Streptococcus	3.95
Parabacteroides	2.28	Prevotella_9	2.81
Ruminococcus	2.20	Odoribacter	2.44
Christensenellaceae_R-7_group	2.14	Faecalibacterium	2.03
Oscillospiraceae_UCG-002	2.07	Lactobacillus	2.01
Subdoligranulum	1.75	Parabacteroides	1.82
Odoribacter	1.70	Izemoplasmatales	1.61
o__Bacilli_RF39	1.70	Erysipelotrichaceae_UCG-003	1.46
Erysipelotrichaceae_UCG-003	1.67	Anaerococcus	1.44
Lachnospiraceae_NK4A136_group	1.65	Lachnospiraceae_NK4A136_group	1.17
Blautia	1.62	Christensenellaceae_R-7_group	0.98
Roseburia	1.52	Subdoligranulum	0.97
Prevotellaceae_NK3B31_group	1.47	[Eubacterium]_siraeum_group	0.90
Paraprevotella	1.22	Roseburia	0.90
Muribaculaceae_CAG-873	1.21	o__Rhodospirillales	0.86
other	26.90	other	19.31

Supplemental table 11. Relative abundances as percentages of genera present in gut microbiota (FE, left) and genera secreting gut microbiota -derived EVs (EV, right) in the colorectal cancer dataset. The 20 most abundant genera have been listed, and the less abundant genera have been combined in the "other" category.

Genus	FE (%)	Genus	EV (%)
Bacteroides	10.43	Faecalibacterium	12.48
Streptococcus	6.31	Prevotella	9.78
Akkermansia	5.93	Bacteroides	8.13
Enterococcus	4.31	Eubacterium_coprostanoligenes_group	4.75
Faecalibacterium	3.97	Ruminococcus	3.64
Prevotella	3.91	Akkermansia	3.51
Alistipes	3.20	Subdoligranulum	2.72
Subdoligranulum	3.08	Romboutsia	2.37
Dialister	2.88	Turicibacter	2.18
Phascolarctobacterium	2.33	Oscillospiraceae_UCG-002	1.98
Bifidobacterium	2.24	Bifidobacterium	1.93
Clostridia_UCG-014	2.12	Enterococcus	1.71
Escherichia-Shigella	2.00	Ruminococcaceae_CAG-352	1.60
Lactobacillus	1.63	Clostridia_UCG-014	1.55
Parabacteroides	1.62	Alistipes	1.47
Ruminococcus_gnavus_group	1.55	Oscillospiraceae_UCG-005	1.46
Eubacterium_coprostanoligenes_group	1.53	Blautia	1.42
Veillonella	1.48	Christensenellaceae_R-7_group	1.41
f__Enterobacteriaceae	1.23	Parabacteroides	1.30
Oscillospiraceae_UCG-002	1.18	Clostridium_sensu_stricto_1	1.12

Supplemental table 12. Read counts of lymphoma dataset samples (all samples).

Sample (Feces)	Read count (Feces)	Sample (EV)	Read count (EV)
F1	7367	E1	9868
		E2	4862
F3	6268	E3	5456
F4	9510	E4	6370
F5	6830	E5	7779
F6	4849	E6	3325
F7	8845	E7	6337
F8	10111	E8	6262
F9	6413	E9	9562
F10	18874	E10	6235
F11	1673	E11	8026
F12	12064	E12	12038
F13	20011	E13	10505
F14	5101	E14	5114
F15	9874	E15	7468
F16	7086	E16	5887
F17	6060	E17	8552
F18	10708	E18	8108
F19	13022	E19	5403
F20	10757	E20	8223
F21	11091	E21	13267
F22	6227	E22	4749
F23	13777	E23	9297
F24	11295	E24	9883
F25	9564	E25	10996
F26	12208	E26	6510
F27	7388	E27	8511

Supplemental table 13. Read counts of Diffuse Large B-Cell lymphoma dataset samples.

Sample (Feces)	Read count (Feces)	Sample (EV)	Read count (EV)
		E1	4862
F2	6268	E2	5456
F3	9510	E3	6370
F4	6830	E4	7779
F5	4849	E5	3325
F6	10111	E6	6262
F7	18874	E7	6235
F8	20011	E8	10505
F9	10708	E9	8108
F10	13022	E10	5403
F11	6227	E11	4749
F12	13777	E12	9297
F13	11295	E13	9883
F14	9564	E14	10996
F15	12208	E15	6510

Supplemental table 14. Read counts of Hodgkin lymphoma dataset samples.

Sample (Feces)	Read count (Feces)	Sample (EV)	Read count (EV)
F1	7367	E1	9868
F2	12064	E2	12038
F3	7086	E3	5887
F4	10757	E4	8223
F5	11091	E5	13267
F6	7388	E6	8511

Supplemental table 15. Read counts of Parkinson's disease dataset samples.

Sample (Feces)	Read count (Feces)	Sample (EV)	Read count (EV)
F1	5949	E1	9378
F2	11195	E2	7693
F3	6841	E3	8819
F4	5652	E4	14374
F5	7454	E5	13612
F6	5552	E6	12082
F7	10336	E7	18646
F8	9010	E8	12768
F9	15853	E9	11313
F10	8373	E10	13514
F11	9880	E11	17895
F12	8767	E12	12288
F13	6695	E13	19210
F14	8447	E14	14835
F15	12340	E15	16409
F16	14580	E16	22373
F17	7478	E17	6797
F18	7520	E18	8294
F19	6858	E19	9055
F20	8966	E20	19281

Supplemental table 16. Read counts of solid tumor dataset samples.

Sample (Feces)	Read count (Feces)	Sample (EV)	Read count (EV)
F1	6434	E1	8979
F2	8498	E2	8759
F3	15497	E3	13755
F4	16573	E4	8091
F5	9720	E5	6950
F6	14278	E6	13634
F7	9482	E7	10684
F8	9528	E8	14680
F9	10322	E9	10151
F10	6861	E10	7879
F11	12493	E11	8152
F12	10552	E12	5497
F13	10663	E13	8069
F14	11000	E14	19133
F15	10812	E15	6108
F16	12122	E16	12979
F17	6941	E17	17203
F18	12628	E18	18952
F19	7803	E19	13196
F20	14183	E20	18364
F21	6835	E21	9882
F22	13016	E22	22516
F23	10656	E23	14354
F24	8246	E24	4083
F25	12995	E25	12654
F26	11078		
F27	9865		

Supplemental table 17. Read counts of pregnancy dataset samples.

Sample (Feces)	Read count (Feces)	Sample (EV)	Read count (EV)
F1	46940	E1	3097
F2	22780	E2	3217
F3	31291	E3	3365
F4	190	E4	3851
F5	63225	E5	3944
F6	25955	E6	4583
F7	58485	E7	3546
F8	36490	E8	6028
F9	28266	E9	4129
F10	20101		
F11	46544	E11	4498
F12	58388	E12	6450
F13	30688	E13	6488
F14	30549	E14	5681
F15	18761	E15	4523
F16	34088	E16	9357
F17	25770	E17	4153
F18	24020	E18	3649
F19	25302	E19	4686
F20	22300	E20	4046
F21	2416	E21	3487
F22	30754	E22	4766
F23	22624	E23	3758

Supplemental table 18. Read counts of obesity dataset samples.

Sample (Feces)	Read count (Feces)	Sample (EV)	Read count (EV)
F1	19047	E1	14028
F2	21171	E2	12272
F3	21269	E3	50201
F4	18438	E4	13102
F5	21172	E5	9315
F6	20651	E6	13242
F7	29951	E7	12790
F8	21955	E8	17051
F9	22009	E9	15135
F10	17954	E10	13255
F11	19882		
F12	18073	E12	12889
F13	20489	E13	13583
F14	20218	E14	20183
F15	24366	E15	12481
F16	26348	E16	20546
F17	19123	E17	16366
F18	20814	E18	20513
F19	23426	E19	8866
F20	15037	E20	53340
F21	21722	E21	15158
F22	23752	E22	2536
F23	26330	E23	17783
F24	22621	E24	12311
F25	24114	E25	13102
F26	27184	E26	15585
F27	31229	E27	13152
F28	22966	E28	20068
F29	36891	E29	7995
F30	21303	E30	12646

Supplemental table 19. Read counts of non-diseased dataset samples.

Sample (Feces)	Read count (Feces)	Sample (EV)	Read count (EV)
F1	32030	E1	14573
		E2	10722
F3	24261	E3	19489
F4	22879	E4	29267
F5	26352	E5	17797
F6	22189	E6	17358
F7	10391	E7	23425
F8	21851	E8	21232
F9	14493	E9	32393
F10	12662	E10	4474
F11	15166	E11	8990
F12	16422	E12	7888
F13	12372	E13	6602
F14	15644	E14	13823
F15	18432	E15	10281
F16	22802	E16	19602
F17	7955	E17	5823
F18	9634	E18	7717
F19	7923	E19	15515
F20	21074	E20	8949
F21	11243	E21	8813
F22	17969	E22	9329
F23	4367	E23	6486
F24	12257	E24	619
F25	8483	E25	13581
F26	12961	E26	10960
F27	4310	E27	11285
F28	8472	E28	13290

Supplemental table 20. Read counts of colorectal cancer dataset samples.

Sample (Feces)	Read count (Feces)	Sample (EV)	Read count (EV)
F1	5958	E1	36987
F2	7015	E2	39227
F3	5213	E3	36011
F4	17990	E4	20224
F5	16102	E5	17724
F6	12735	E6	13298
F7	17621	E7	11934
F8	17658	E8	11969
F9	15315	E9	18600
F10	7990	E10	21543
F11	11216	E11	19709
F12	13127	E12	15959
F13	9064	E13	23566
F14	4785	E14	23031
F15	20076	E15	15665
F16	17442	E16	20514
F17	12791	E17	13683
F18	10716	E18	16784
F19	14132	E19	19475
F20	13917	E20	14277
F21	11507	E21	12943
F22	10501	E22	17714
F23	9700	E23	15014
F24	10520	E24	6397
F25	6324	E25	27077
F26	9078	E26	10907
F27	14315	E27	10641
F28	36850	E28	38728
F29	30688	E29	15685
F30	47183	E30	29148
F31	7868	E31	8406
F32	34168	E32	8589
F33	9536	E33	14088
F34	11706	E34	11572
F35	11991	E35	2972
F36	7501	E36	26986
F37	5871	E37	19968
F38	23405	E38	17214
F39	19460	E39	9741
F40	28157	E40	9740
F41	18556	E41	16583
F42	14897	E42	726

F43	22557	E43	20958
F44	26553	E44	8766
F45	19245	E45	49566
F46	38609	E46	17506
F47	6531	E47	35512
F48	28278	E48	34715
F49	28642	E49	61813
F50	24413	E50	29084
F51	8146	E51	74731
F52	34208	E52	24409
F53	16649	E53	80521
F54	20774	E54	35004
F55	36940	E55	11674
F56	34319	E56	14842
F57	33465	E57	19574
F58	7018	E58	35212
F59	30216	E59	19133
F60	32991	E60	20418
F61	28118	E61	16083
F62	32720	E62	16796
F63	14348	E63	41444
F64	12097	E64	30588
F65	13064	E65	39696
F66	68574	E66	18809
F67	51716	E67	26626
F68	7582	E68	26969
F69	5804	E69	34420
F70	7433	E70	6025

Supplemental table 21. Details on how sequences were processed in each dataset.

Dataset	Min. raw read length	Denoising program	Pooling method for denoising	Chimera removal method	Read length after denoising	Decontamination program	N of samples	N of reads	N of features
Lymphoma	200	DADA2	Independent	Consensus	330	Decontam	53	455,556	321
Parkinson's disease	200	DADA2	Independent	Consensus	330	Decontam	40	446,382	348
Solid tumor	200	DADA2	Independent	Consensus	330	Decontam	52	583,785	347
Pregnancy	200	DADA2	Independent	Consensus	265	Decontam	46	807,229	428
Obesity	200	DADA2	Independent	Consensus	230	Decontam	59	1,158,999	636
Non-diseased	200	DADA2	Independent	Consensus	330 (disease controls) 230 (obesity controls)	Decontam	55	784,877	566
CRC	200	DADA2	Independent	Consensus	fwd: 270 rev: 185	None	140	2,913,568	946
